# Binding-driven reactivity attenuation enables NMR identification of selective drug candidates for nucleic acid targets

**DOI:** 10.1038/s42004-022-00755-8

**Published:** 2022-10-27

**Authors:** Laura Díaz-Casado, Andrés G. Santana, Irene Gómez-Pinto, Alejandro Villacampa, Francisco Corzana, Jesús Jiménez-Barbero, Carlos González, Juan Luis Asensio

**Affiliations:** 1 Instituto de Química Orgánica (IQOG-CSIC), Juan de la Cierva 3, 28006 Madrid, Spain; 2 Instituto de Química-Física Rocasolano (IQFR-CSIC), Madrid, 28006 Spain; 3 Dept. Química and Centro de Investigación en Síntesis Química, Universidad de La Rioja, 26005 La Rioja, Spain; 4 Center for Cooperative Research in Biosciences (CIC-bioGUNE). Derio, 48160 Bizkaia, Spain

**Keywords:** Screening, Nucleic acids, Drug discovery and development

## Abstract

NMR methods, and in particular ligand-based approaches, are among the most robust and reliable alternatives for binding detection and consequently, they have become highly popular in the context of hit identification and drug discovery. However, when dealing with DNA/RNA targets, these techniques face limitations that have precluded widespread application in medicinal chemistry. In order to expand the arsenal of spectroscopic tools for binding detection and to overcome the existing difficulties, herein we explore the scope and limitations of a strategy that makes use of a binding indicator previously unexploited by NMR: the perturbation of the ligand reactivity caused by complex formation. The obtained results indicate that ligand reactivity can be utilised to reveal association processes and identify the best binders within mixtures of significant complexity, providing a conceptually different reactivity-based alternative within NMR screening methods.

## Abstract (web summary)

NMR spectroscopy could be a robust and reliable method for binding detection in drug discovery, but DNA/RNA targets remain challenging to analyze. Here, the authors explore the perturbation of the ligand reactivity caused by complex formation as a binding indicator to identify best binders within mixtures of significant complexity, providing a conceptually different reactivity-based alternative within NMR screening methods.

## Introduction

The specific association of low-molecular-weight ligands to DNA/RNA fragments are molecular recognition events of capital importance in biological and medicinal chemistry. Indeed, nucleic acids are direct targets for most cytotoxic drugs, which are still extensively used in the treatment of a wide range of human cancers^1^. Moreover, RNA has emerged as a key regulatory element in multiple biological processes. Although only a 3% of the human genome codes for proteins, around 85% is transcribed into RNA. A large number of these noncoding RNAs are involved in diseases, both in cancer and non-tumorigenic disorders, providing a wealth of promising and previously unrecognised therapeutic targets^2^.

**Fig. 1:**
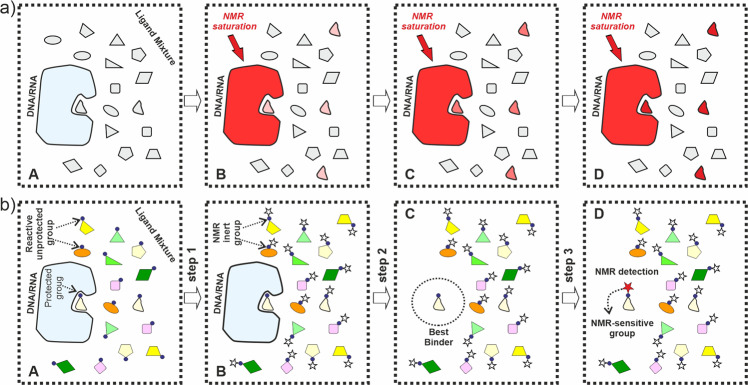
STD vs reactivity-based NMR screening. **a** Schematic representation of the STD-NMR experiment commonly used for the identification of binders. Upon saturation of the receptor signals, magnetisation is transferred to the ligands. **b** Concept figure illustrating a feasible strategy for a reactivity-based screening protocol. The expected decrease in ligand reactivity associated to complex formation is exploited to preferentially label the best binders within a mixture of candidates.

NMR spectroscopy has been extensively employed, for more than three decades now, to detect and characterise binding processes of biomedical relevance, making use, in most cases, of changes in chemical shift, relaxation times, diffusion constants, NOEs (nuclear Overhauser effects), or exchange of saturation. Indeed, a variety of strategies have been introduced over the time, many of which have found widespread applications in both academic research and pharmaceutical industry^3–16^. Among them, ligand-based methodologies have gained popularity in recent years as they are especially suitable for small binders of weak-to-medium affinity, no isotopic labelling of the receptor is required and upper limits for its size are virtually non-existent. In addition, most of these methods can be easily integrated in screening protocols as they are fast and relatively inexpensive. Not surprisingly, ligand-based NMR strategies have become highly popular in the context of hit identification and drug discovery. Unfortunately, despite its undeniable versatility and numerous advantages, NMR also faces some limitations as a detection tool, some of which are especially severe for nucleic acids. First, non-specific association of cationic ligands to the poly-anionic surface of the targets represents a common and serious problem. Furthermore, the short correlation time inherent to the typically employed medium-sized DNA/RNA fragments, together with their reduced proton density and limited chemical shift dispersion as compared to proteins, pose additional challenges for STD-based (saturation transfer difference spectroscopy; Fig. 1a) or tr-NOE (transferred nuclear Overhauser effect) screening methods^17^.

In order to address these issues, herein we put forward an alternative approach, which is based on a fundamentally different binding indicator unexploited by current screening methodologies: the limitation in the ligand reactivity promoted by complex formation (herein referred to as ligand protection). This effect has been shown for the molecular recognition of aminoglycoside antibiotics by RNA aptamers in the context of synthetic applications, and would be expected to be general for different receptors or reactions^18^. In principle, changes in the ligand reactivity associated to binding could be revealed employing the simple strategy represented in Fig. 1b. First, reactive centres of the ligand mixture are partially modified by the attachment of an “NMR-inactive” moiety (step 1). This chemical reaction is expected to affect mainly unprotected, weak and non-specific binders with respect to those buried within the binding-pocket, effectively deleting (from an NMR perspective) these components from the sample mixture. Secondly, the receptor is removed (i.e., by enzymatic digestion) (step 2), prior to selective labelling of the best binders, employing this time an NMR-active reporter group (Step 3). Finally, reaction mixtures are submitted to NMR analysis to reveal the best binders. In contrast with common approaches, this reactivity-based screening would be expected to distinguish between superficial binders (attached to the receptor polyphosphate backbone by means of weak electrostatic interactions) and those specifically recognised by the receptor binding-pocket. Herein, we explore the scope and limitations of this strategy.

## Results

The general concept illustrated in Fig. 1b could be materialised through a variety of reactions/labels and NMR experiments, depending on the chemical nature of the compound libraries. Considering that polyamine scaffolds are relatively common among nucleic acid binders, we decided to exploit the distinct reactivity of –NH_2_ groups with reductive amination reactions. Among the different possible fragments to attach at these sites, methyl groups present several characteristic advantages from an NMR perspective: they comprise three equivalent protons, typically produce sharp NMR signals and, if properly labelled with ^13^C, they can be detected even at low micromolar concentrations employing HSQC-type (heteronuclear single quantum coherence) experiments. Taking this into account, N-^12^CH_3_ and N-^13^CH_3_ methyl groups (introduced through reductive amination reactions with formaldehyde) were selected as NMR-inactive and NMR-active functions, respectively (see also Supplementary Fig. S1).

The following discussion is structured into three sub-sections. First, we focused our attention on an important family of bio-active RNA binders, the aminoglycoside antibiotics^19–23^. Thus, the decrease in the aminoglycoside reactivity promoted by an RNA receptor, in the context of the selected reaction is demonstrated and quantified, employing a simple NMR assay set up in our group (protocol 1, see below). Second, the sensitivity of the observed protection effects to the ligand/receptor complementarity and the strength of the existing intermolecular contacts is briefly discussed. Finally, protection effects are implemented in actual screening assays, employing an adapted version of the general strategy shown in Fig. 1b (protocol 2). This strategy has been tested with a variety of aminated derivatives and both RNA and DNA fragments with distinct architectures.

### Molecular recognition and ligand reactivity: defining protection factors

**Fig. 2:**
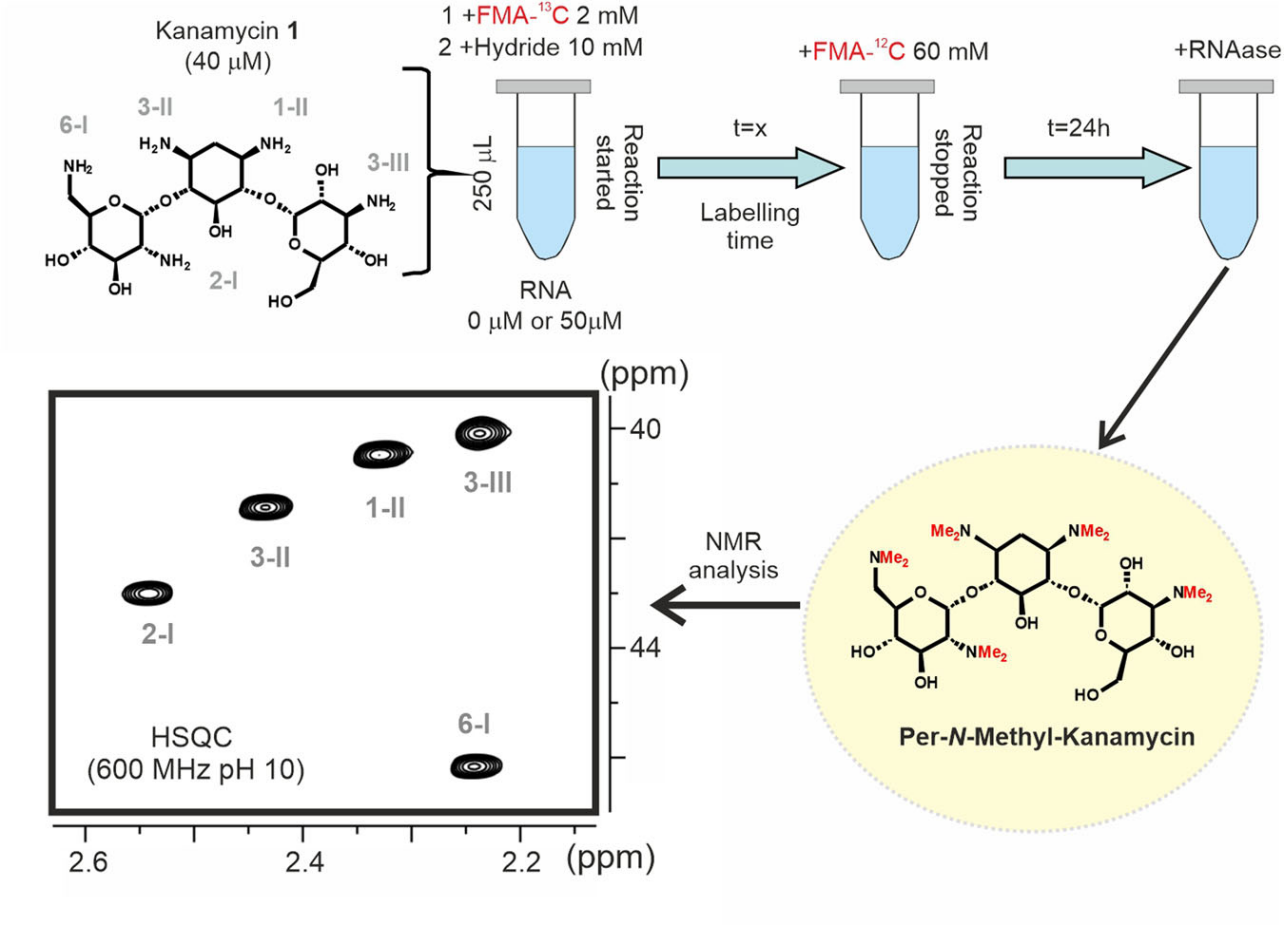
Experimental protocol to measure protection factors. Schematic representation of the strategy employed to derive *protection factors* (protocol 1). *N*-Methylation reactions of kanamycin-B (**1**) with ^13^C-formaldehyde (FMA) were carried out with both the free (-RNA) and complexed (+RNA) ligand. After a variable labelling time (*t* = x), aliquots were taken and treated with a large excess of unlabelled reagent to yield a single per-*N*-methyl derivative. Finally, RNA receptor is digested and the samples submitted to HSQC NMR analysis (spectrum shown corresponds to the resulting per-*N*-methyl-kanamycin at pH 10.0, where the assignment is also indicated).

**Fig. 3:**
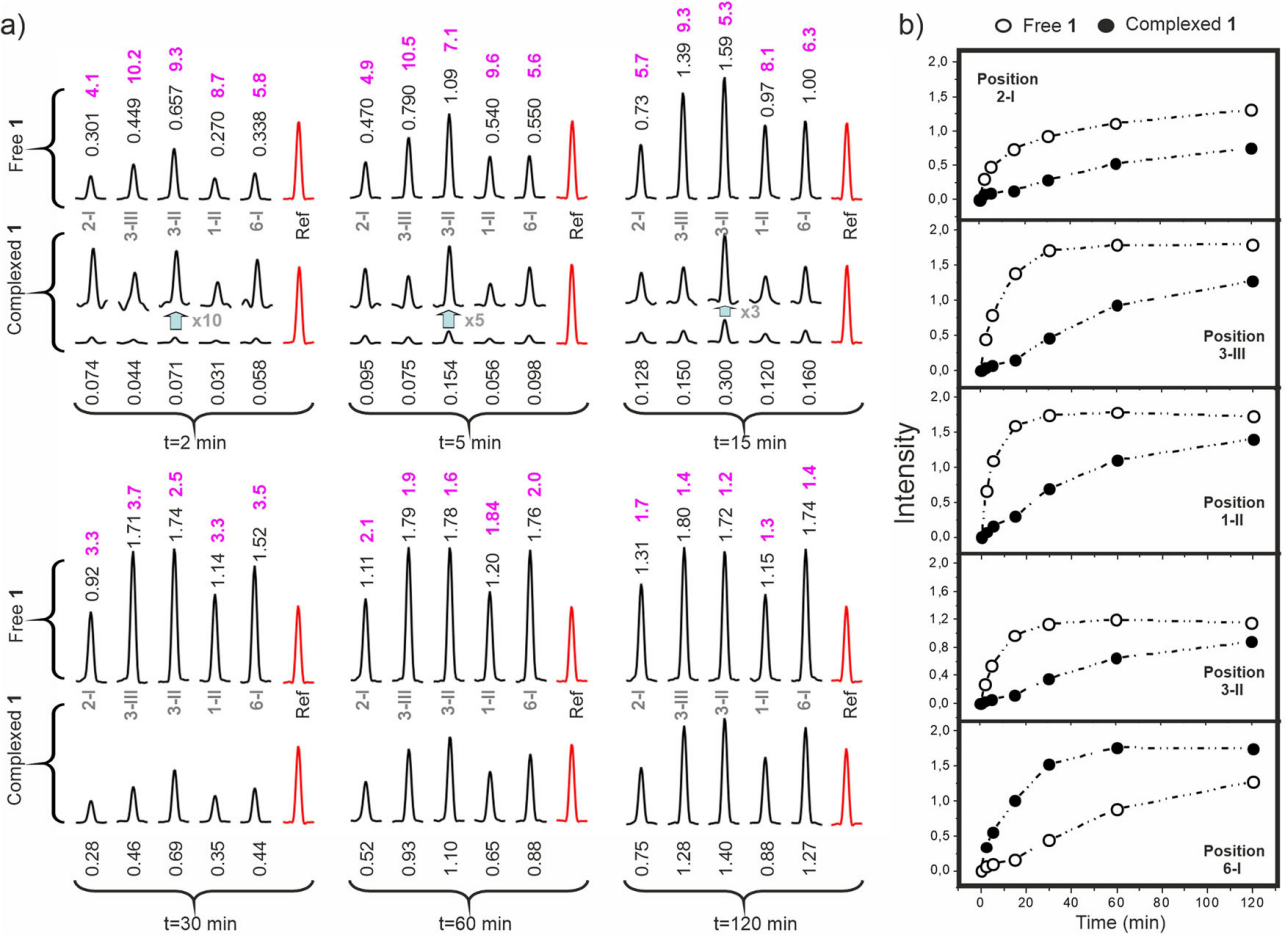
Reactivity of kanamycin complexed to the A-Site. **a** Cross sections for the five HSQC cross-peaks at different labelling times (*t* = x), both in the absence (above) and presence (below) of the RNA receptor (for short reaction times, these latter peaks have also been scaled up for clarity). The corresponding intensity ratios are shown in magenta. **b** Intensity build-up curves for the five HSQC cross-peaks measured in the absence and presence of RNA.

A central hypothesis to this work is that association with the nucleic acid receptor prevents, to some extent, the ligand from accessing to the selected reagent, thus decreasing its apparent reactivity. In order to test this point, kanamycin-B (**1**) was employed as a model compound and its *N*-methylation kinetics was analysed, both in the absence and presence of the wild-type ribosomal A-site RNA fragment (wt-RNA)^24–30^. This can be carried out following the simple NMR-based protocol represented in Fig. 2 and Supplementary Fig. S2 (protocol 1). Two buffered solutions containing free and complexed **1** (40 μM each) are subjected to a reductive amination reaction with ^13^C-labelled formaldehyde (FMA-^13^C, 2 mM). This isotopically labelled reagent allows the NMR detection of the reaction products with large sensitivity and for this reason it will be used throughout the manuscript for screening purposes. However, in this section it will be employed just for monitorization of the reaction kinetics. The reaction is triggered by the addition of sodium cyanoborohydride and then left to proceed for a certain labelling-time (2 min–24 h), after which a large excess of unlabelled formaldehyde (>50 mM) was added to complete the reaction, thus yielding a sole per-*N*-methyl kanamycin derivative. Finally, the RNA receptor was enzymatically digested and both ligand solutions were transferred to NMR tubes for analysis. As shown in Fig. 2, HSQC spectra acquired from these samples (neutral compound at pH > 10.0) are extremely simple, showing just five peaks (one per -NMe_2_ group), whose absolute intensities depend on the fraction of ^13^CH_3_ incorporated at every ligand reactive position during the labelling period. Integration of NMR cross-peaks in spectra obtained with increasing labelling-times allowed for the monitorization of the full reductive amination kinetics for each particular amine moiety (see the experimental section). The obtained results are represented in Fig. 3 and Supplementary Fig. S3. As expected, complex formation promotes a significant decrease in the ligand apparent reactivity. For every reactive position, a protection factor parameter (Pf) was defined as the ratio between the initial reaction rates measured in the absence and presence of the wt-RNA receptor. The obtained values are represented in Fig. 3, ranging from 4.1 for position 2-I of the drug to a 10.2-fold reactivity drop for the amino group 3-III. These differences result from a variety of factors, such as the accessibility of the different amino moieties in the complex, their participation in polar interactions with RNA and/or changes in their protonation state associated to complex formation.

### Dissecting protection factors: binding strength and ligand accessibility

**Fig. 4:**
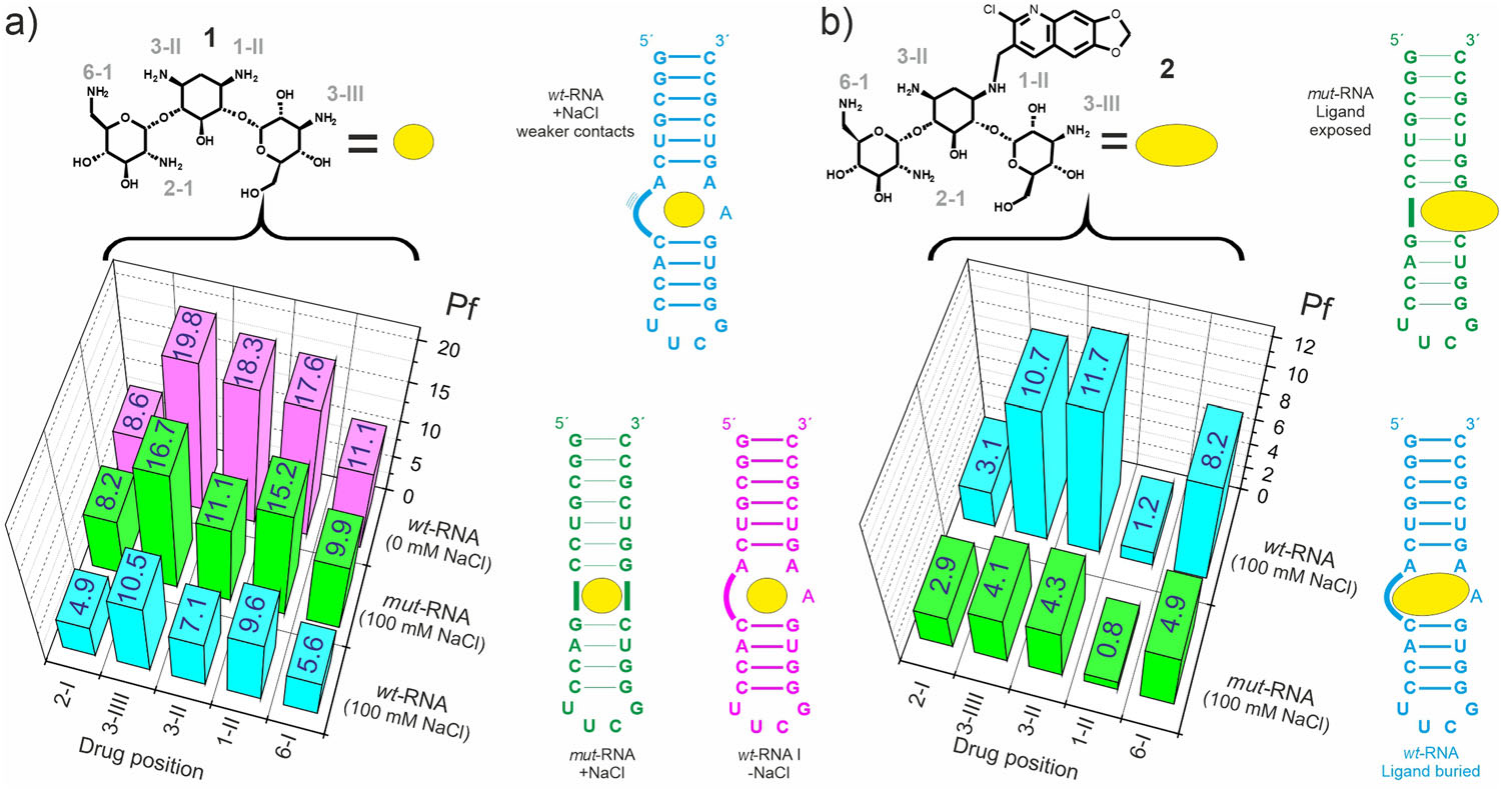
Reactivity of aminoglycoside/RNA complexes. **a** Protection factors determined for **1**, complexed to *wt-*RNA and *mut-*RNA (green) in independent assays. In the tests employing the *wt-*RNA, the drug reactivity was measured both at 0 and 100 mM NaCl (magenta and cyan, respectively). **b** Protection factors determined for **2** complexed to *wt-*RNA (cyan) and *mut-*RNA (green) in independent assays. In all cases, ligand and RNA concentrations were 40 μM and 50 μM, respectively.

In contrast with more conventional binding indicators, protection factors would be expected to be sensitive to the buried/exposed character of the bound ligand and also to the strength of its contacts with the receptor, which in turn may provide an idea of how intimate the interaction is within the binding pocket. The decrease in the ligand reactivity associated to complex formation was measured under low ionic strength (0 mM NaCl). It is well established that this experimental set up enhances ligand/receptor polar contacts, leading to a significant increase in the complex stability. Indeed, in the absence of any ionic competitors, polar interactions mediated by the ligand –NH_3_^+^ groups and the receptor phosphates should be stronger, exhibiting longer residence times and eventually leading to a sharper decrease in the ligand reactivity. The obtained results were fully consistent with this view (Fig. 4a). Thus, while the reactivity profile measured at 0 mM NaCl (Fig. 4a, magenta) is similar to that previously described at 100 mM NaCl (which could be taken as indicative of a common binding mode), individual protection factors are, on average, two-fold larger, ranging from 8.6 to a staggering 19.8, almost a twenty-fold decrease in reactivity for that individual amino group (3-III). Mg^2+^-containing electrolytes, such as MgCl_2_, would be expected to exert a similar influence on the reactivity of the complexed ligand to that observed for NaCl.

As a next step, we tested the ligand protection with a mutated A-site RNA variant (mut-RNA. Fig. 4a). According to previous results by our group^31^, this mut-RNA fragment binds kanamycin-B (**1**) with similar affinity to the wild-type target. However, being devoid of an internal loop motif, it displays a narrower major groove leading to a presumably less exposed binding region. In agreement with our expectations, the mut-RNA receptor promotes a sharper decrease in the kanamycin-B reactivity, whereas the drug reactivity profile is basically maintained (Fig. 4a and Supplementary Figs. S4 and S5).

Lastly, instead of changing the size of the receptor binding-pocket we modified the ligand dimensions through a chemical modification. Kanamycin derivative **2**, tethered with an aromatic fragment, has been recently identified by us from a combinatorial screening protocol and, by virtue of its larger volume, displays a clear preference for the wild-type A-site fragment rather than towards the mut-RNA variant^31^. To our delight, this selectivity is clearly reflected in the measured protection factors. Indeed, in contrast with the behaviour exhibited by the natural aminoglycoside **1**, compound **2** is more significantly protected by the wider internal loop present in wt-RNA. On the contrary, the bulkier dimensions of **2** effectively prevent penetration into the major groove of the mut-RNA variant, leading to a more superficial, less protecting interaction mode (Fig. 4b).

### Revealing selective binding by ligand protection: towards a reactivity-based screening

**Fig. 5:**
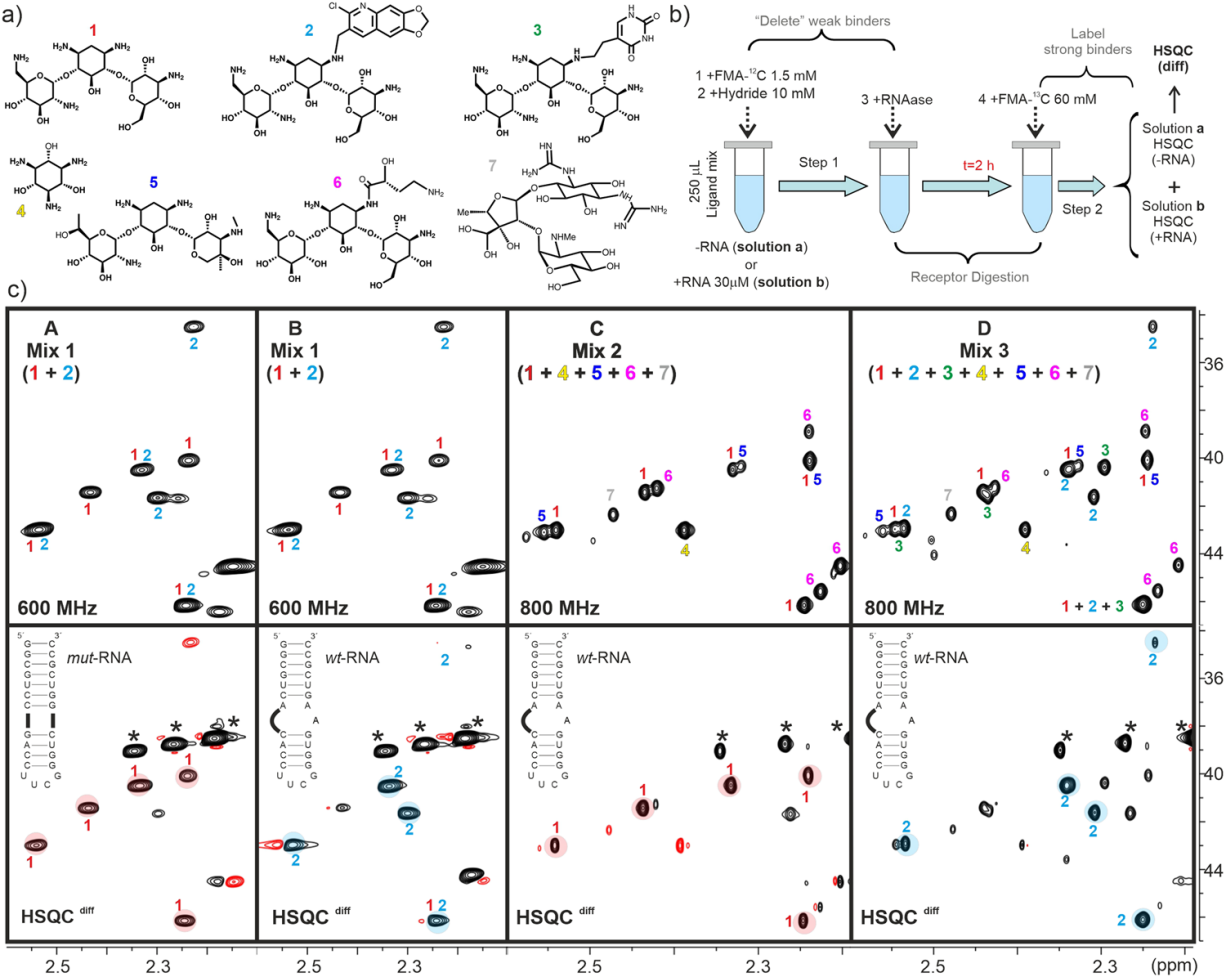
Protection assays with aminoglycoside mixtures. **a** RNA ligands employed for the different assays. **b** Schematic representation of the reactivity-based screening protocol (protocol 2). Ligand mixtures of increasing complexity, both in the absence or presence of 30 μM receptor (–RNA and +RNA samples, respectively), were treated with 1 mM unlabelled formaldehyde under reductive amination conditions. After RNA digestion, the reaction is completed with an excess of labelled reagent. The resulting mixtures of per-*N*-methyl-ligands were analysed by means of HSQC experiments at pH 10 (neutral species). In order to highlight differences in cross-peak intensity between the HSQC^+RNA^ and HSQC^−RNA^ data sets, they were subtracted to provide an HSQC^diff^ spectrum. **c** HSQC^−RNA^ (top) and HSQC^diff^ (bottom) data sets obtained after applying our protocol to ligand *mixtures 1*-*3* (from left to right). The RNA receptor employed in every case is shown. Cross-peaks for every mixture component are labelled. HSQC^diff^ spectra (bottom) allow the identification of the best binders.

Next we tested the potential of ligand reactivity as a binding indicator in screening protocols, employing relatively simple mixtures of cationic derivatives (derivatives **1**–**7**, Fig. 5a) and the previously described RNA targets. For comparison purposes, the performance of common STD/trNOESY approaches were also assayed (Supplementary Fig. S6). A serious limitation of STD experiments becomes apparent when selecting the ligand libraries; the reduced signal dispersion characteristic of RNA targets, which must be selectively saturated, precluded us from including ligands with aromatic protons in our mixtures. Taking this into account, a simple library formed by three aminoglycosides (**1** + **5** + **7**) was selected, spanning around >20-fold range in binding affinity for the A-site target (reported K_b_ values for **1** and **5** are 0.7–1.1 10^6^ M^−1^ and 5.4 10^4^, respectively consistent with a difference in total charge of +1). Streptomycin **7**, which has a different ribosome target, binds to the A-site non-specifically with K_b_ < 10^4^ M^−1^^31–33^. As shown in Supplementary Fig. S6, only few small STD effects (in most cases below 1%) can be measured without interference from the saturated RNA signals (implementation of T2 filters does not alleviate this problem). On the other hand, the three ligands exhibit clear negative NOEs in trNOESY experiments. While both data sets correctly point to **7** as the weakest ligand, the identification of **1** as the best binder is far more problematic. Non-specific association of the three cationic compounds to the RNA target (even at 0.2 M NaCl) render both relaxation-based approaches ambiguous and inconclusive. In our opinion, most of these limitations could be overcome by complementing chemical relaxation with chemical reactivity as a binding indicator.

For screening purposes, the general concept illustrated in Fig. 1b was adapted as represented in Fig. 5b (protocol 2). According to this strategy, two solutions containing identical ligand mixtures but in the absence or presence of RNA, were subjected to a partial *N*-methylation reaction, employing unlabelled formaldehyde. Then, the receptor was digested by treatment with RNAase. Finally, both solutions were treated with ^13^C-labelled formaldehyde under reductive amination conditions to mark the unreacted amino groups in the mixtures of per-*N*-methyl-derivatives, which were analysed by means of HSQC experiments. NMR spectra obtained from both reaction mixtures (herein referred to as HSQC^−RNA^ and HSQC^+RNA^) contain an identical number of signals. However, in the latter case cross-peak intensities are biased in favour of the stronger binders, which results from the differential protective effect exerted by the receptor during step 1; the best binders are somewhat sheltered from the unlabelled reagent, incorporating larger fractions of ^13^CH_3_ after the RNA digestion (see Figs. 1b and 5b).

In summary, differences in intensity between HSQC^−RNA^ and HSQC^+RNA^ reveal the protective influence of the RNA and can be employed to point out the strongest binders. Thus, HSQC^+RNA^ and HSQC^−RNA^ can be subtracted to produce a simplified difference spectrum (HSQC^diff^), which displays positive signals only for the best ligand. In order to evaluate differences in cross-peaks intensity and to facilitate an accurate subtraction of the HSQC data sets, ^13^C-sodium acetate, present at identical concentrations in all the NMR samples, was employed as internal reference.

We carried out several assays with mixtures of increasing complexity (from 2 to 7 ligands. Fig. 5c and Supplementary Figs. S7–S12). We started with a simple two-component solution formed by aminoglycosides **1** and **2** (mix 1). Fig. 5c shows the HSQC^−RNA^ (top) and HSQC^diff^ (bottom) data sets obtained after applying our protocol to the binary mixture, either with the mutated or the wild-type RNA receptors (panel A or B, respectively). It can be observed that, in contrast to the HSQC^−RNA^ experiments, HSQC^diff^ spectra seem to reflect the presence of a preponderant mixture component, allowing a straightforward identification of the best binder. As expected, the wt-RNA fragment exerts a preferential protection of ligand **2** (Fig. 5c, panel B), whereas ligand **1** is selectively protected by the mutated variant (Fig. 5c, panel A). This observation is fully consistent with the protection factors described for the alternative ligand/receptor pairs in the previous section.

Further experiments performed with wt-RNA receptor and ligand solutions containing 5 (mix 2) and 7 (mix 3) aminoglycoside compounds (Fig. 5c, panels C and D, respectively) confirmed the potential of ligand protection to evaluate association processes. Indeed, despite the significant signal overlapping displayed by the HSQC^−RNA^ experiments, difference data sets permitted, in both cases, to single out the best binders (derivatives **1** and **2** in mixtures 2 and 3, respectively).

**Fig. 6:**
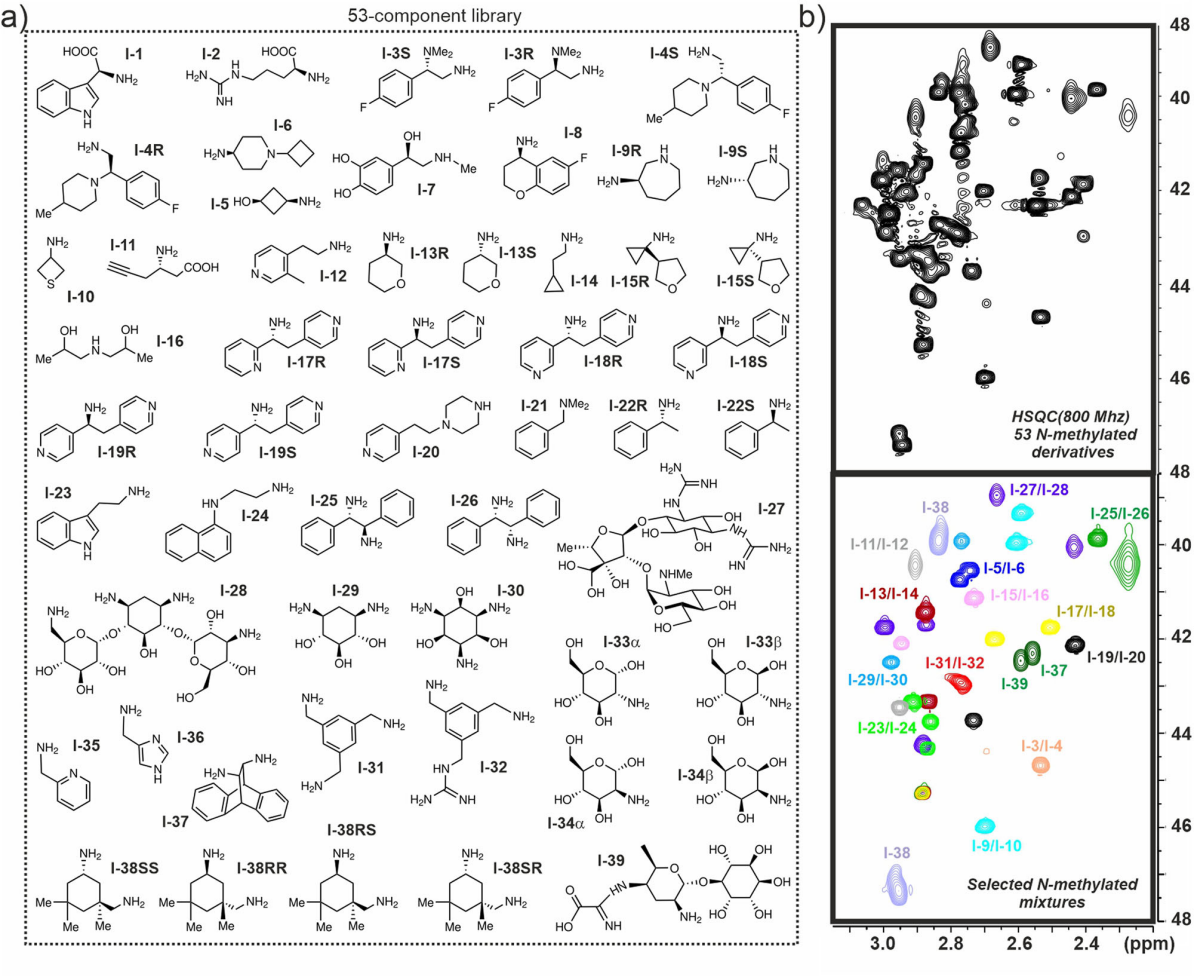
Library of aminated derivatives. **a** Library of 53 mono- and polyaminated derivatives employed in our chemical protection assays. **b** HSQC experiment (pH 7.2) obtained for the ^13^C- per-N-methylated library (top) together with a superimposition of HSQC data sets measured for selected pairs of per-N-methylated components for assignment purposes (bottom).

**Fig. 7:**
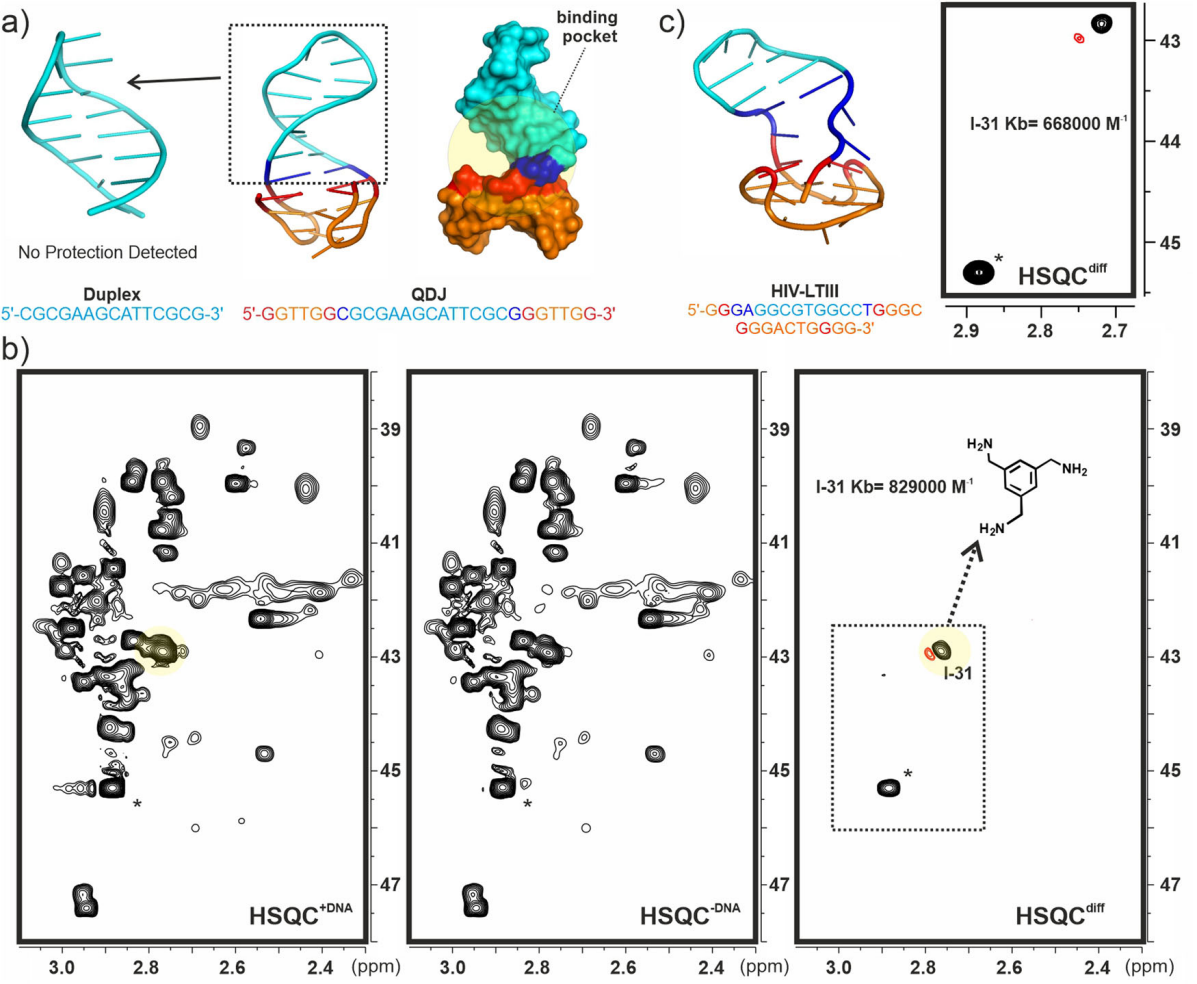
Protection assays with DNA quadruplex-duplex junctions. **a** Structure of the simple B-DNA duplex and model quadruplex-duplex junction (QDJ) employed in our assays. **b** Protection experiments performed employing the complete 53-compound library in the presence of the QDJ fragment. HSQC^+DNA^, HSQC^−DNA^ and HSQC^diff^ data sets (pH 7.2) are represented. **c** Protection experiment performed with the HIV-LTIII fragment (above). A section of the resulting HSQC^diff^ data set is represented.

Encouraged by these positive results, we decided to test our approach in a more realistic screening scenario. Thus, we built a library of 53 aminated and poly-aminated derivatives of different sizes, comprising total charges in the +1/+4 range (Fig. 6a derivatives **l-1** to **l-39**). For particular components we included alternative enantiomers/stereoisomers. As expected, the resulting mixture produced extremely overlapped 1D-NMR spectra which rendered the sample intractable by conventional NMR-based screening approaches. Fortunately, the observed complexity is partially reduced in the 2D-HSQC data-set measured for the ^13^C-permethylated sample (Fig. 6b). This mixture was employed in chemical protection assays analogous to those previously described, with a selection of DNA fragments. Preliminary assays with a simple B-DNA duplex (Fig. 7a and Supplementary Fig. S13) showed no significant protection for any of the library components. Taking these into account, we focused our attention on DNA topologies exhibiting clear bulges or potentially occluded binding pockets. The model quadruplex-duplex junction recently described by T. Phan and co-workers and displayed in Fig. 7a represents a clear example of this topology^34^. Indeed, close inspection of its 3D structure reveals the existence of a deep cavity located just at the duplex-quadruplex interface. In principle, association of low molecular weight molecules to this pocket would be expected to have a significant protecting influence on the ligands, rendering the junction motif ideally suited for reactivity-based NMR screening methods. Fittingly, previous studies by our group have revealed that simple benzylamine fragments bind selectively to quadruplex-duplex junctions by inserting an ammonium group at the centre of the interfacial G-tetrad. In fact, according to ITC measurements, library components **l-31** and **l-32** display binding affinities in the 10^5 ^M^−1^ range for this DNA target^35^.

For comparison purposes we acquired first an STD-NMR data-set employing a simple mixture formed by 10 derivatives (Supplementary Fig. S14). This experiment was consistent with the simultaneous occurrence of several binding processes, some of which might reflect weak non-specific electrostatic associations to the DNA surface. On the contrary, protection assays carried out with the whole library (Fig. 7b and Supplementary Fig. S15) revealed that just one component gets substantially protected by the junction architecture. Indeed, the HSQC^diff^ data set displayed two unique cross-peaks, one of them corresponding to the per-methylated derivative **l-31** while the accompanying signal, marked with an asterisk in the figure, is formed upon methylation of ammonia present as a minor contaminant in the DNA samples.

Ligand **l-31** has also been shown to be a selective binder of a related, but much more relevant, quadruplex-duplex junction occurring in the LTR-III region of the HIV-1 virus^36^. Satisfactorily, reactivity assays performed with this receptor employing our protocol again confirmed **l-31** as the library component showing the most significant protection by the DNA receptor (minor effects were also detected for **I-28**. See the HSQC^diff^ spectra in Fig. 7c and Supplementary Fig. S16).

**Fig. 8:**
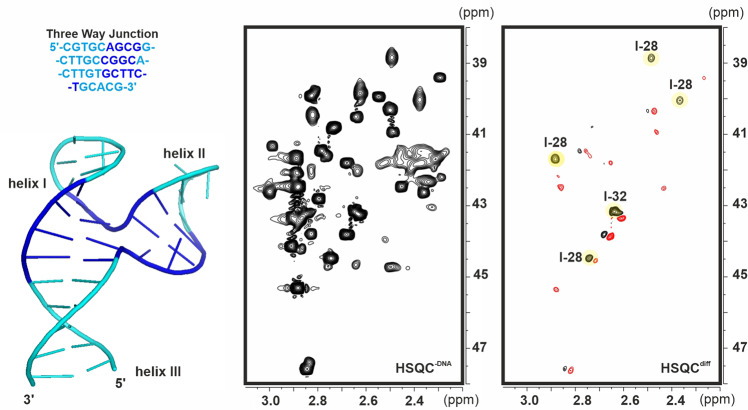
Protection assays with a Three-way junction DNA. Structure of the model three-way junction (TWJ) employed in our assays together with the protection experiments performed employing the complete 53-compounds library. HSQC^−DNA^, and HSQC^diff^ (imperfect subtraction due to minor pH differences between samples confers an antiphase look to some signals) data sets are represented on the middle and right panels respectively (pH 7.4).

Finally, we focused our attention on a model three-way junction DNA described by Wijmenga and col^37^. According to NMR data this structure displays potentially occluded regions at the confluence between the three duplex stems and therefore represents an adequate model for our purposes (Fig. 8). As previously observed for the quadruplex-duplex junctions, STD experiments performed with a very simple mixture formed by only six compounds, revealed several binding events involving derivatives **l-27**, **l-28**, **l-30** and **l-39** (Supplementary Fig. S17). In contrast, according to protection assays carried out with the complete library, only 2 derivatives (**l-28** and **l-32**) get protected by the DNA fragment, reflecting their association at relatively buried sites of the junction structure (Fig. 8). This point was further confirmed by independent NMR titration experiments which showed that both ligands bind to a common region involving residues T31 and G7 at the confluence between helical stems II and III (Supplementary Fig. S18) with significant affinity. Attempts were made to estimate the stability of the complexes by means of NMR titrations. These experiments showed that saturation of the receptor at 50 μM DNA were superior to 80% and 90% for 1:1 and 2:1 ligand/DNA ratios, respectively, which allowed us to stablish a lower limit of K_b_ > 200000 M^−1^ for both ligands. Titrations performed with alternative ligand mixtures under identical experimental conditions detected no significant association of any other component in agreement with the reactivity assays.

In summary, the protocol herein reported permits the detection of reactivity changes associated to the formation of true ligand/receptor complexes involving nucleic acids and can be implemented in screening protocols with convoluted mixtures of aminated ligands. Our approach provides complementary information to that derived by more conventional STD-NMR or trNOE experiments, while overcoming some of the associated limitations.

## Conclusions

Herein we propose an alternative NMR-based approach for the detection and characterisation of binding events involving nucleic acid targets. In contrast with previously described methods, our strategy makes use of NMR to reveal changes in the ligand reactivity associated to complex formation. Such reactivity-based screening protocol presents several advantages: first, no upper limit exists for the binding constants. Second, assays can be run at small scale, employing reduced amounts of ligands and receptors. Indeed, according to tests performed by us, ligand protection can be detected even with receptor and ligand concentrations in the low micromolar range. Third, the reported protocol shows a reduced sensitivity to the main adjustable experimental parameters, namely the concentrations of ligands, RNA/DNA and unlabelled reagent employed during the first deleting step, thus providing reliable and reproducible data. Fourth, protection assays are relatively fast and can be easily parallelised for screening purposes. Fifth, this protocol overcomes most of the limitations of conventional STD/tr-NOE strategies, particularly those derived from the reduced proton density and signal dispersion characteristic of nucleic acid fragments. Sixth, and most importantly, in contrast to other binding indicators, the reactivity of the complexed ligand is exquisitely sensitive to the environment provided by the receptor. Specifically, while superficial binding (either promoted by hydrophobic or electrostatic interactions) has a significant influence on most NMR parameters, this interaction mode has a limited effect on ligand reactivity. In this regard, it is important to note that under the employed experimental conditions, the RNA/DNA receptors interact with the components of the ligand libraries in multiple ways, which comprise both strong and weak (non-specific) binding events. Indeed, multiple binding represents a common feature of aminoglycoside/RNA association processes as revealed by ITC studies^38–40^. Regardless of the complexity of the resulting scenario, ligand protection provides a simple means to identify reactivity-limiting complexes, thus ruling out superficial association modes.

As a final point, it should be noted that the proposed methodology could be equally employed to reveal catalysis by the receptor, an uncommon but not implausible scenario. Thus, according to our protocol, complexed ligands “protected” by the DNA/RNA from the unlabelled formaldehyde will incorporate larger fractions of the ^13^C-labelled reagent after the receptor digestion, showing larger cross-peaks in the HSQC^+DNA/RNA^ spectrum. This is the most common situation and the behaviour observed in all the assays included in the manuscript. Conversely, catalysis by the DNA/RNA fragment would translate in an enhanced incorporation of unlabelled *N*-methyl groups, implying that the corresponding signals would display significantly reduced intensities in the HSQC^+DNA/RNA^ experiment. In summary, any change in reactivity, either inhibition or enhancement, could be equally revealed by comparing the HSQC^+DNA/RNA^ and HSQC^−DNA/RNA^ data sets. These reactivity perturbations could also be taken as evidence of binding for screening purposes.

To conclude, the obtained results validate the general strategy proposed in this study and set the basis for a conceptually different type of reactivity-based NMR screening methods.

## Methods

The 27-mer A-site RNA fragment (receptor wt-RNA) together with a mutated version without internal loop (receptor mut-RNA) were prepared by in vitro transcription as previously described^41, 42^. DNA oligonucleotides 5′-CGCGAAGCATTCGCG-3′ (for the duplex structure), 5′-GGTTGGCGCGAAGCATTCGCGGGTTGG-3′ (for the model quadruplex-duplex junction, QDJ1), 5′-GGGAGGCGTGGCCTGGGCGGGACTGGGG-3′ (for the HIV-LTIII quadruplex-duplex junction) and 5′-CGTGCAGCGGCTTGCCGGCACTTGTGCTTCTGCACG-3′ (for the three-way junction structure, TWJ) were purchased in its purified desalted form from IDT (Integrated DNA technologies). Both, RNA and DNA oligos were dialysed in the appropriate buffer. RNA assays were performed in 100 mM NaCl, 10 mM Na_2_PO_4_ buffer at pH 7.5. For the DNA duplex and quadruplex-duplex junction structure we employed 20 mM KCl, 20 mM K_2_HPO_4_ at pH 7.0. Finally, experiments with the three-way junction structure (TWJ) were performed in 50 mM KCl, 10 mM K_2_HPO_4_ at pH 6.5.

To ensure a proper folding of the different RNA or DNA structures the corresponding oligonucleotides were subjected to an annealing protocol. With this purpose they were placed in a water bath at 293 K, heated to 358 K for 5 min and then slowly cooled back down to 293 K over a 2 h period.

Compounds **1**, **4**–**7** and library derivatives **l-1** to **l-39** (except **I-31** and **I-32**) together with both unlabelled and ^13^C-labelled formaldehyde samples were purchase from Sigma-Aldrich. Kanamycin derivatives **2–3**, **I-31** and **I-32** were prepared by our group as recently reported^31, 35^.

### NMR-based analysis of aminoglycoside N-Methylation Kinetics in the free and RNA-complexed states (protocol 1)

Ligand solutions (V = 250 μL each) containing the free and RNA-complexed ligand were prepared in 100 mM NaCl, 10 mM Na_2_PO_4_ buffer at pH 7.5. For the complexed state, lyophilised RNA samples were dissolved in buffer and renatured as previously outlined. Then, ligands **1** or **2** were added from stock solutions. Final aminoglycoside concentration in both solutions was 40 μM, while for the complexed state we employed a slight excess of the RNA fragment (50 μM). According to the literature, binding affinities of **1** and **2** for the employed receptors (wt-RNA or mut-RNA) are in the 10^6^–10^8 ^M^−1^ range, implying that under the employed experimental conditions, the fraction of ligand bound to RNA was, in all cases, larger than 95%^31–33, 38–40^. As a next step, both solutions (see Fig. 1) were treated with 2 mM ^13^C-formaldehyde. The reaction was triggered with 10 mM sodium cyanoborohydride, and then left to proceed for a certain labelling-time at 20 °C, before adding a significant excess of ^12^C-formaldehyde (final concentration 50 mM). The objective of this isotopic dilution step was to stop the incorporation of ^13^CH_3_ groups to the aminoglycoside, yielding a single per-N-methyl derivative. Next, we digested the RNA fragment with RNAase-A (1 μM, 2 h) and ligand solutions were transferred to NMR tubes for analysis. The fraction of ^13^C- incorporated at each reactive drug position was evaluated by means of HSQC experiments acquired at pH 10.0. HSQC cross-peaks for per-N-methyl- **1** and **2** derivatives have been previously assigned by means of HMBC spectra^31, 43^ and were integrated employing ^13^C-sodium acetate, present in the reaction buffer, as internal reference (100 μM in both samples). This process was repeated employing growing labelling times, ranging from 2 min to 24 h to derive the full reaction curve for every drug reactive position. A protection factor (Pf) was defined for all of them as the ratio between initial reaction rates in the absence and presence of RNA. Uncertainties in their values fall within the following ranges: ±5% for 1 < Pf < 5, ±10% for 5 < Pf < 10 and ±20% for 10 < Pf < 20. Protection factors for compound **1** were derived under a variety of experimental conditions including low ionic strength or acidic pH.

### Reactivity-based screening assays (protocol 2)

Solutions containing the different ligand mixtures assayed in this work were prepared in the adequate buffer (see above) both in the absence and presence of the nucleic acid receptor. For binary ligand mixtures, total ligand and receptor concentrations were fixed at 100 μM and 30 μM, respectively. For the more complex libraries (containing 5, 7 or 53 compounds), total ligand concentrations were in the 300–1000 μM range while that of the nucleic acids was maintained at 30 μM. As a first step in our screening protocol (step 1 in Fig. 5b) we carried out a reductive amination reaction on both solutions, employing ^12^C-formaldehyde and 10 mM sodium cyanoborohydride. Regarding the formaldehyde, different concentrations, in the 0.5–2 mM, were tested depending on the complexity of the ligand mixture. Next, the RNA or DNA fragment is digested with RNAase-A (1 μM, 2 h) or DNAase, respectively. Finally (step 2 in Fig. 5b), we carried out a second reductive amination reaction, employing 20 mM ^13^C-formaldehyde and 30 mM sodium cyanoborohydride. After 12 h, reaction mixtures were transferred to NMR tubes and analysed. HSQC experiments were acquired from both samples (herein referred to as HSQC^−RNA/DNA^ and HSQC^+RNA/DNA^, respectively) in Bruker Avance 600 MHz or Bruker Avance 800 MHz spectrometers equipped with cryo-probes. Resolved signals in HSQC^+RNA/DNA^ and HSQC^−RNA/DNA^ data sets were integrated employing as internal reference ^13^C-sodium acetate, present in the reaction buffer (100 μM). Intensity ratios were taken as indicative of the receptor protective influence. Alternatively, HSQC^+RNA/DNA^ and HSQC^−RNA/DNA^ spectra were subtracted to produce a difference HSQC data set (herein referred to as HSQC^diff^), which allows a straightforward identification of the protected mixture components.

### STD and trNOESY experiments

NMR spectra were acquired with a Bruker Avance 600 MHz spectrometer at 293 K. NMR samples were prepared in the adequate buffer (see above), employing 50 μM concentration for the nucleic acid and 1–2 mM for the individual ligands. STD data sets were acquired with 1024 scans, using a train of 50 ms Gaussian-shaped pulses for the selective saturation of the RNA/DNA protons at *δ* = 7.54 or 5.6 ppm. The total saturation time was fixed to 2 s and the off-resonance frequency was set at *δ* = 100 ppm. No water-suppression scheme was applied. Test assays were also performed employing a T1 rho spin-lock filter to suppress the RNA signals. trNOESY experiments in the presence of the A-Site RNA were carried out employing 200 ms mixing time. Control data sets with no RNA revealed the presence of weakly positive or null NOEs for all the mixture components. On the contrary, all the mixture components (Figure S6) exhibited clear negative NOEs in the presence of the A-site as a result of the association process.

## Supplementary information

Asensio_PR File

Supplementary Information

## Data Availability

The authors declare that all data supporting the findings of this study are available within the article and its supplementary information files, and from the corresponding author on reasonable request. Comments about the relationship between protection factors and binding affinity an experimental section detailing theoretical simulations performed with simple protection experiments and Supplementary Figs. S1–S22 are provided as supporting information.
