# Supplementary material for: Binding-driven reactivity attenuation enables NMR identification of selective drug candidates for nucleic acid targets
Source: Commun Chem. 2022 Oct 27;5:137. doi: 10.1038/s42004-022-00755-8 (PMC9814457; doi:10.1038/s42004-022-00755-8)
Supplement: Supplementary file 2 — Supplementary Information [file 42004_2022_755_MOESM2_ESM.pdf]

Supporting Information

For

Binding-driven reactivity attenuation enables NMR identification of selective drug candidates for nucleic acid targets

*Laura Díaz-Casado,[1] Andrés G. Santana,[1] Irene Gómez-Pinto,[2] Alejandro Villacampa,[1] Francisco Corzana,[3] Jesús Jiménez-Barbero,[4] Carlos González,[2] Juan Luis Asensio[1]**

[1]Instituto de Química Orgánica (IQOG-CSIC), Juan de la Cierva 3, 28006 Madrid, Spain.
[2]Instituto de Química-Física Rocasolano (IQFR-CSIC). Madrid 28006, Spain.
[3]Dept. Química and Centro de Investigación en Síntesis Química, Universidad de La Rioja. 26005, La Rioja, Spain.
[4]Center for Cooperative Research in Biosciences (CIC-bioGUNE). Derio 48160, Bizkaia, Spain

CONTENTS:

Comments about the relationship between protection factors and binding affinity

Supplementary experimental section: Theoretical simulations of the protection assays

Supplementary Figures S1 to S22

Relationship between Protection factors and binding affinity.- Protection factors reflect how much the ligand reactivity its attenuated upon association to a given receptor. Understandably, it is a complex parameter which depends on a variety of factors, the most evident being the accessibility of the reactive groups in the bound state. This dependency implies that protection factors are sensitive to the topology of the receptor binding site (buried or superficial) and the orientation of the ligand. Dynamic fluctuations of the complex might also play an important modulatory role facilitating or hindering the encounter between reactive species. Moreover, direct involvement of the reactive ligand functions in strong inter-molecular interactions, (i.e. hydrogen bonds, salt-bridges, cation/π bonds etc) would be expected to exert a largely inhibitory influence on its reactivity. Notwithstanding, subtler effects could also be at play. For example, reductive amination reactions are sensitive to the protonation state of the amino groups, which are usually significantly altered as a result of complex formation. Indeed, pKa perturbations of ligand groups have been reported for several RNA complexes. Even changes in solvation could also contribute to the observed reactivity alterations.

However, despite the inherent complexity of the topic, a significant part of the factors contributing to ligand protection is inevitably connected with the ligand affinity. Thus, buried/concave "more protecting" binding pockets provide an increased surface for stablishing ligand-receptor contacts than superficial/planar sites, and frequently present more strongly negative electrostatic potentials which would translate into more stable associations. Similarly, enhanced intermolecular interactions produce more stable but also less dynamic complexes which could further inhibit the ligand reactivity. Finally, participation of the ligand reactive functions in strong interactions with the DNA/RNA fragment should reciprocate in both increased protection factors and binding affinities.

Taken all these points together into consideration, protection factors and complex stability would be expected to be, to some extent, correlated. This correlation is nicely illustrated by the association of kanamycin to the A-site fragment at high and low ionic strengths (represented in Fig. 4a) where, having both complexes identical geometry (as proved by the protection factor profiles) the most stable complex was also significantly less reactive. Further evidence for the mentioned correlation is the fact that all the assays described in the manuscript succeeded in identifying the best binder within the employed libraries.

Supplementary experimental section

Numerical simulations with the program GEPASY.- We carried out simulations of the protection experiments employing the biochemical kinetic simulator GEPASY.[1] Simulated assays comprised a DNA/RNA receptor, an *N*-methylating reagent and simple libraries formed by 2-5 derivatives, with binding affinities, in 0-10^6 M^{-1} range. Concentrations for the ligands, and receptor were set to 50 μM and 30 μM, respectively. Regarding the *N*-methylating agent, its concentration was set to 100 μM, for assays with only two derivatives (Supplementary Figures S19-S20), or 300 μM for those assays involving the five component libraries (Supplementary Figures S21-S22). Considering that the chemical modification of the ligands usually proceeds for several hours, this process was taken as slow on the DNA/RNA complexation time scale. Similarly, taking into account that a significant part of the *N*-methylating reagent, formaldehyde, is reduced to methanol under the employed conditions, this secondary reaction pathway was also implemented in the model, being its kinetics empirically adjusted so that roughly 50% on the reagent participates in *productive N*-methylation reactions. Finally, complexed ligands were assumed to be either fully protected by the nucleic acid receptor (Supplementary Figure S19), or to present different protection factors in the 2.5-5 range (Supplementary Figures S20-S22), as indicated. In all cases several simplifying assumptions were made. First, ligands were assumed to have a single reaction site which can incorporate a single *N*-methyl group. Second, reaction rates were considered identical for all the library components. Third, *N*-methylated derivatives were taken as non-binders.

On this basis, numeric integration of the corresponding kinetic equations allowed a theoretical evaluation of the final concentrations for the ^{12}C-*N*-methly-derivatives **^{12}C-ML1-^{12}C-ML5**, from which the concentrations of the ^{13}C-*N*-methly-derivatives **^{13}C-ML1-^{13}C-ML5**, generated in the final methylation step (that is, after the enzymatic digestion of the receptor) were derived (i.e. **^{13}C-ML1**= 50-**^{12}C-ML1**). These values were taken as proportional to the cross-peaks displayed in the HSQC $HSQC^{+DNA/RNA}$ spectra. Cross-peaks in the $HSQC^{-DNA/RNA}$ data set were evaluated by running a second simulation, this time with the receptor concentration set to 0 μM. Relative intensities for calculated $HSQC^{+DNA/RNA}$ and $HSQC^{-DNA/RNA}$ cross-peaks for each simulation are represented at the bottom of Figures S19-S22.

1.- Mendes, P. Biochemistry by numbers: simulation of biochemical pathways with Gepasi 3. *Trends Biochem. Sci.* **22**, 361−363 (1997).

Supplementary Figure S1.- Schematic representation of the reactivity-based screening protocol proposed for poly-amine nucleic acid binders. First, reactive amino centers of weak binders are saturated with $^{12}CH_3$ groups. Upon digestion of the receptor, the best binders are preferentially labeled with $-^{13}CH_3$ groups and identified by NMR.

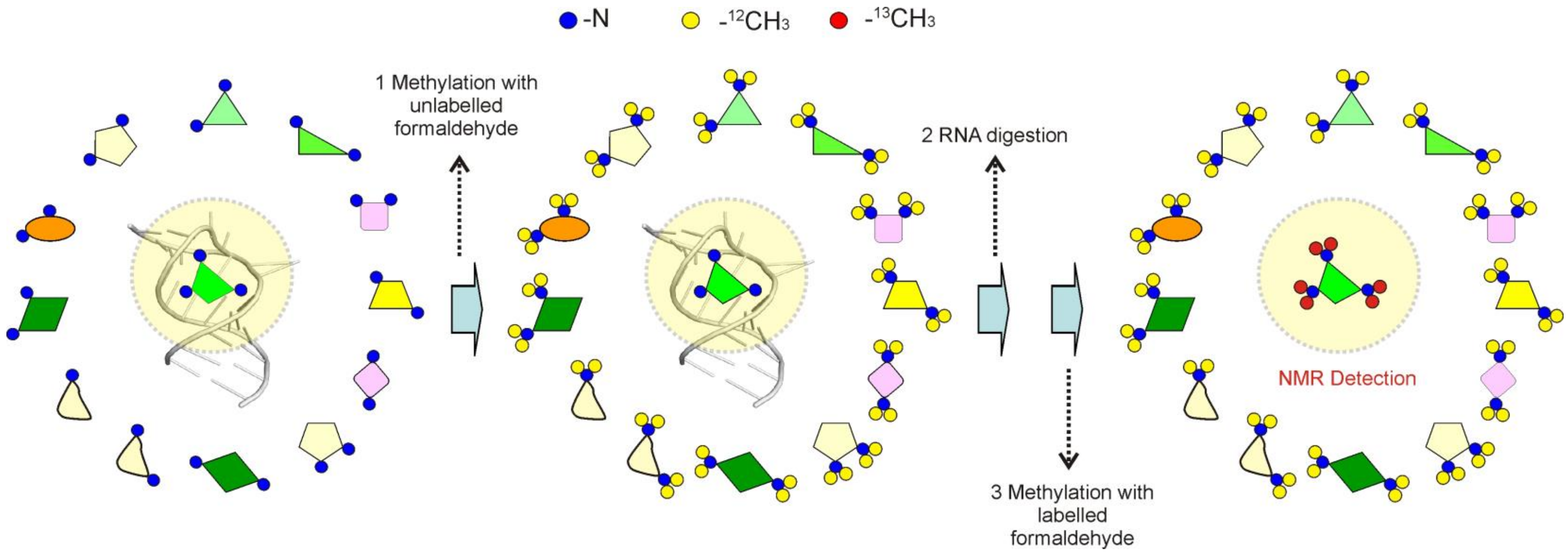

Supplementary Figure S2.- Schematic representation of the strategy employed to analyze the aminoglycoside *N*-methylation kinetics in both the free (up) and complexed (down) states (*protocol 1*). Reaction is started with ^{13}C-formaldehyde (FMA). After a variable *labeling time* (t=x) a large excess of unlabeled reagent is added to yield a single per-*N*-methyl derivative. Finally, RNA receptor is digested and the samples transferred to NMR tubes for analysis. Cross-peaks in the obtained HSQC spectra are proportional to the fraction of ^{13}C incorporated to every reactive position during the labeling time.

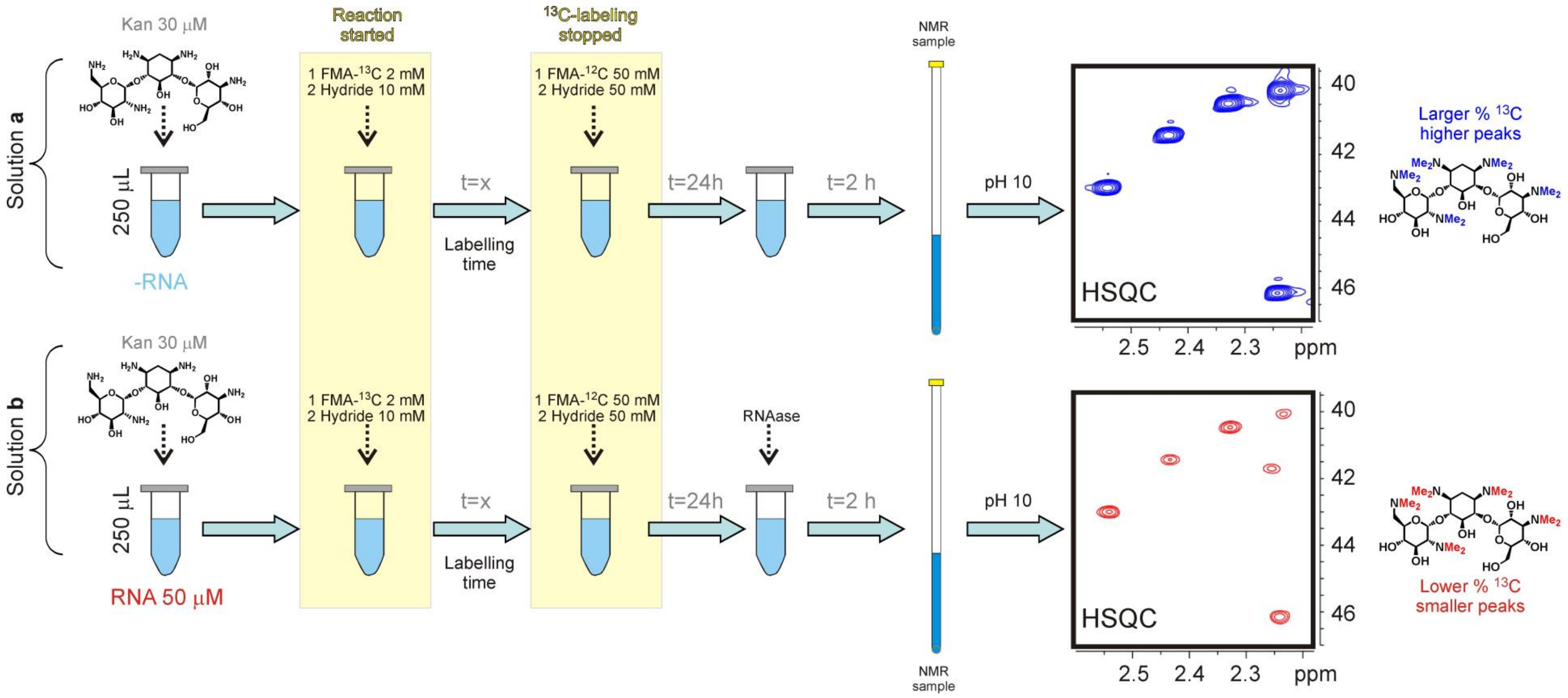

Supplementary Figure S3.- Kanamycin *N*-methylation kinetic experiments performed in the presence and absence of the ribosomal A-Site RNA at pH 7.5 (up) and 5.0 (down). HSQC experiments obtained after applying our protocol (protocol 1; see the experimental section) in the absence and presence of the receptor are represented in blue and red, respectively. The employed labeling time was, in all cases 5 minutes. Cross-sections for the five cross peaks in these HSQC experiments are represented on the right. The corresponding intensity ratios (herein referred to as *protection factors*) are shown in magenta. Notation employed for the different rings and reactive positions are shown on the top.

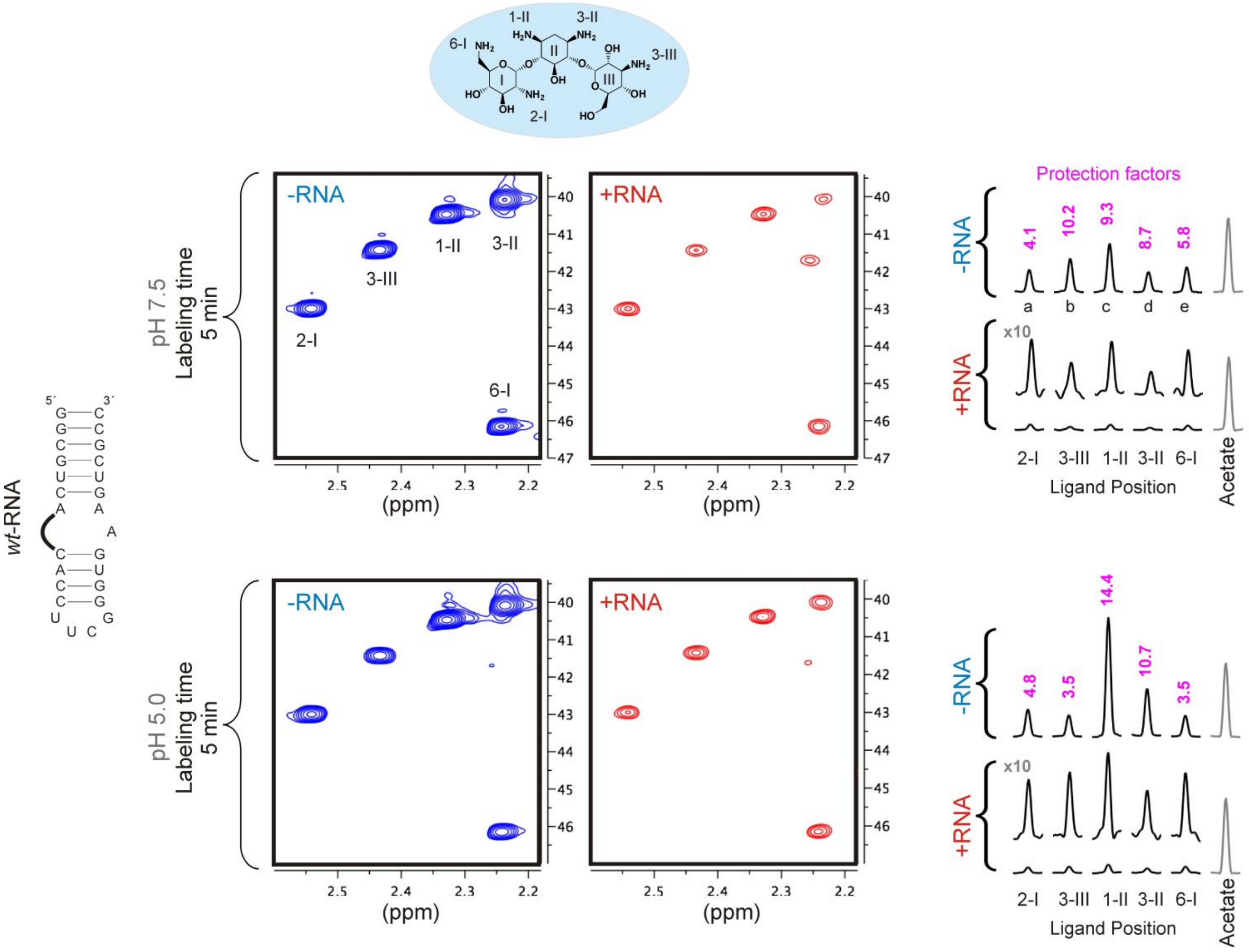

Supplementary Figure S4.- Kanamycin *N*-methylation kinetic experiments performed in the presence and absence of *mut*-RNA (a mutated version of the ribosomal A-Site) at pH 7.5. HSQC experiments (protocol 1; see the experimental section) in the absence (up) and presence (down) of the receptor are represented. The employed labeling time was, in all cases 5 minutes. Cross-sections for the five cross peaks in these HSQC experiments are represented on the right. The corresponding intensity ratios (herein referred to as *protection factors*) are shown in magenta. Notation employed for the different rings and reactive positions are shown on the left.

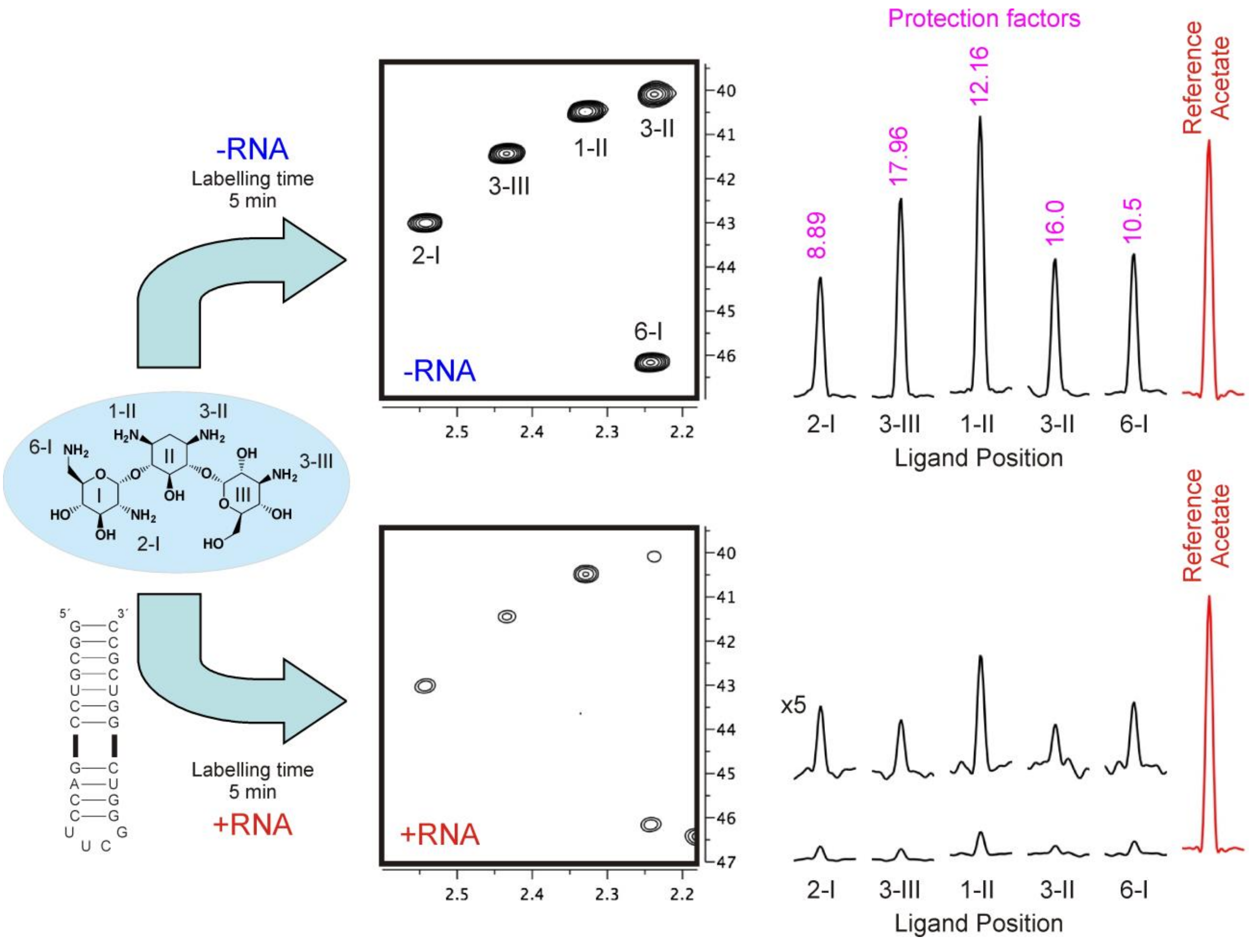

Supplementary Figure S5.- Kanamycin *N*-methylation kinetic experiments performed in the absence and presence of receptor *wt*-RNA (left) or *mut*-RNA (right) at pH 7.5. The employed labeling time was, in all cases 5 minutes. Cross-sections for the five HSQC cross peaks in $HSQC^{+RNA}$ and $HSQC^{-RNA}$ data sets are represented. The corresponding intensity ratios (herein referred to as *protection factors*) are shown in magenta. The notation employed for the different drug units and reactive positions are shown in the center of the Figure.

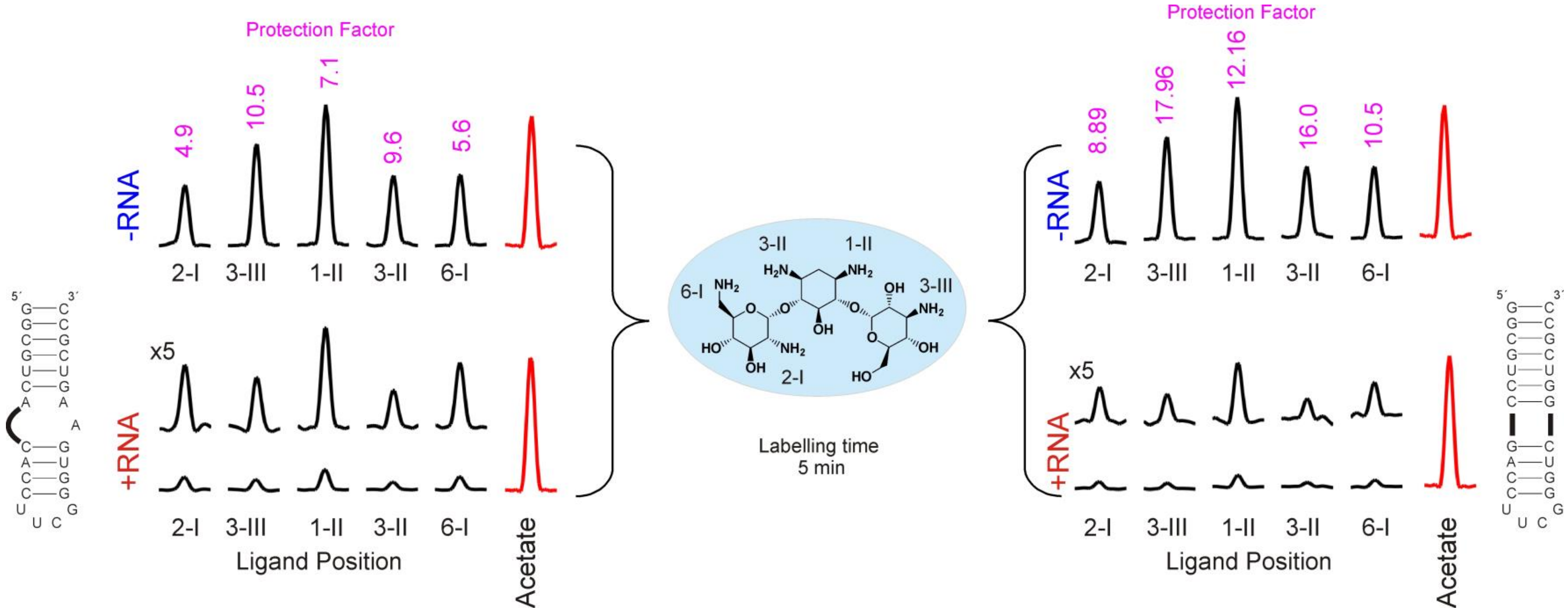

Supplementary Figure S6.- Top.-RNA ligands employed for the STD and trNOESY assays. Bottom-Left Left.- STD NMR spectrum obtained upon saturating (2 s) the target A-site (50 μM) at 7.54 ppm in the presence of ligands **1**, **5** and **7** (1 mM each). A reference spectrum of the mixture is shown below. Bottom-Right.- trNOESY experiment (anomeric region) measured with the same sample employing a mixing time of 0.2 s.

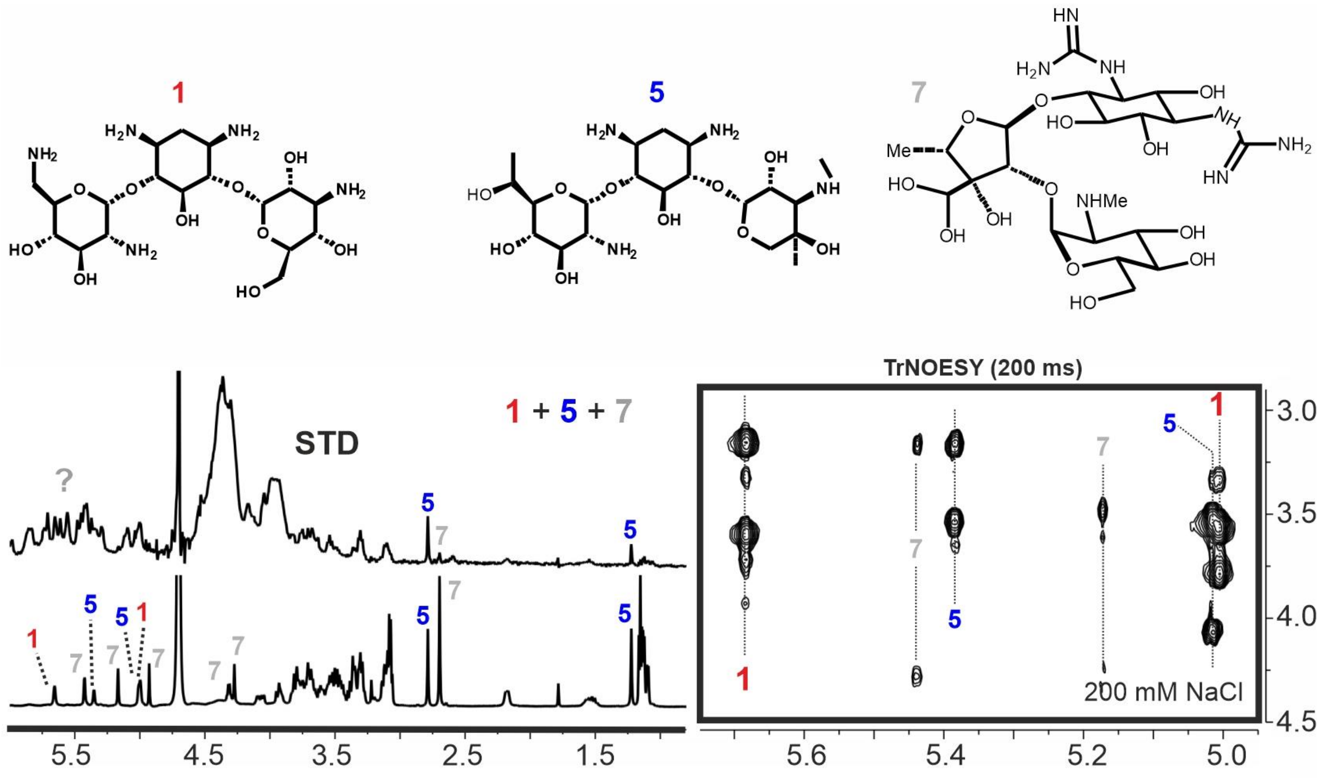

Supplementary Figure S7.- Reference HSQC experiment acquired for a per-*N*-methylated mixture of five polycationic ligands. Mixture components (before methylation), together with their concentrations are shown. Cross peaks are assigned to the different *N*-methylated ligands according to the color code. Check

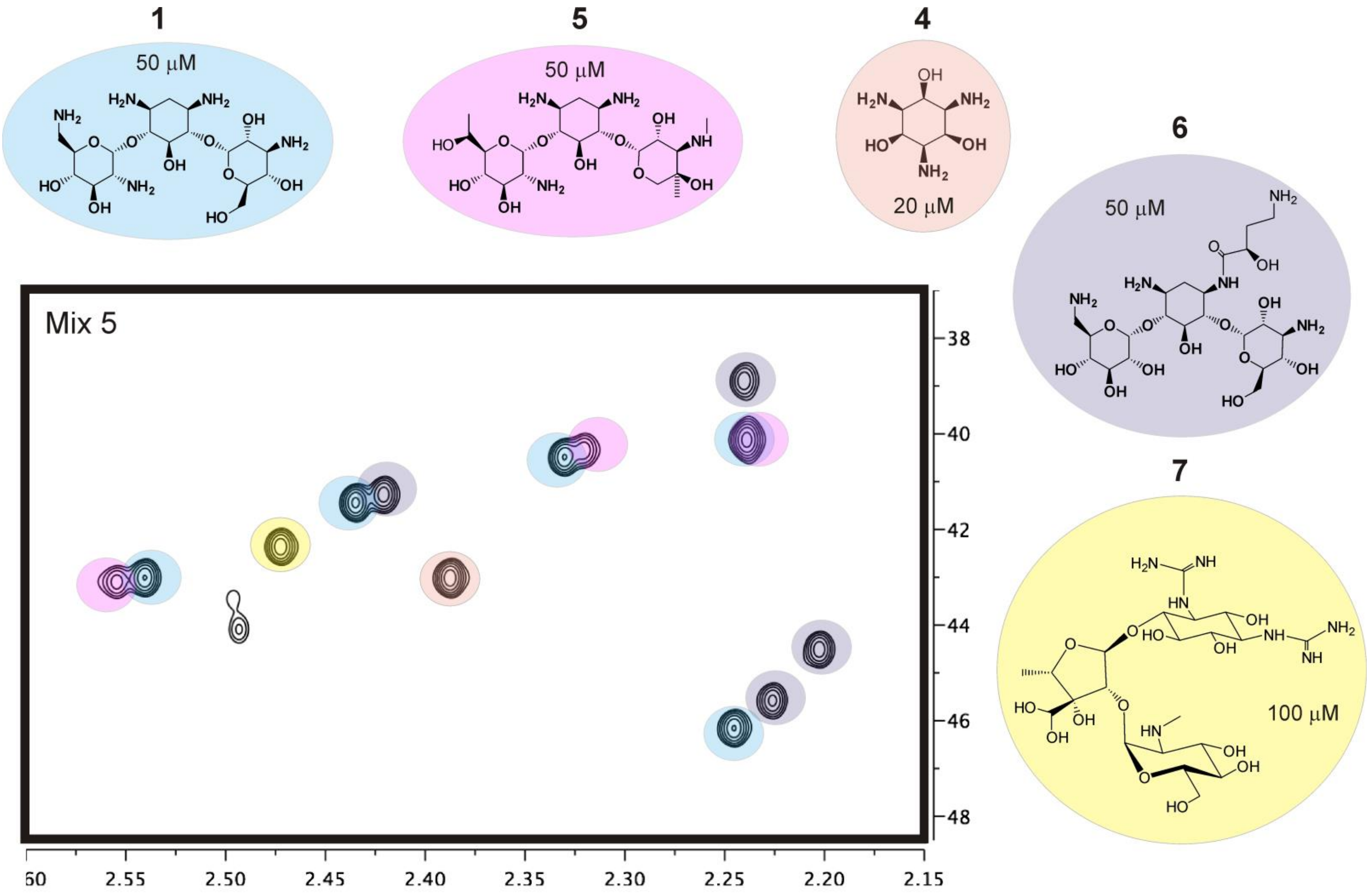

Supplementary Figure S8.- HSQC^{+RNA} and HSQC^{-RNA} experiments (in red and blue, respectively) acquired after applying the proposed screening protocol to a mixture of five polyamine ligands (mix 5), employing the ribosomal A-site RNA as receptor. A reference HSQC data set measured for the per-*N*-methylated mixture is shown on the top (in black). Cross sections for two selected peaks (from per-*N*-methylated ligands **1** and **4**) in these experiments are shown on the left, together with those corresponding to the acetate signal employed as reference for integrals (on the top). It can be observed that, for ligand **1** cross peak intensity in HSQC^{+RNA} (in red) is higher than in HSQC^{-RNA} (in blue), reflecting the protection of this compound by the RNA receptor. The opposite is true for derivative **4**.

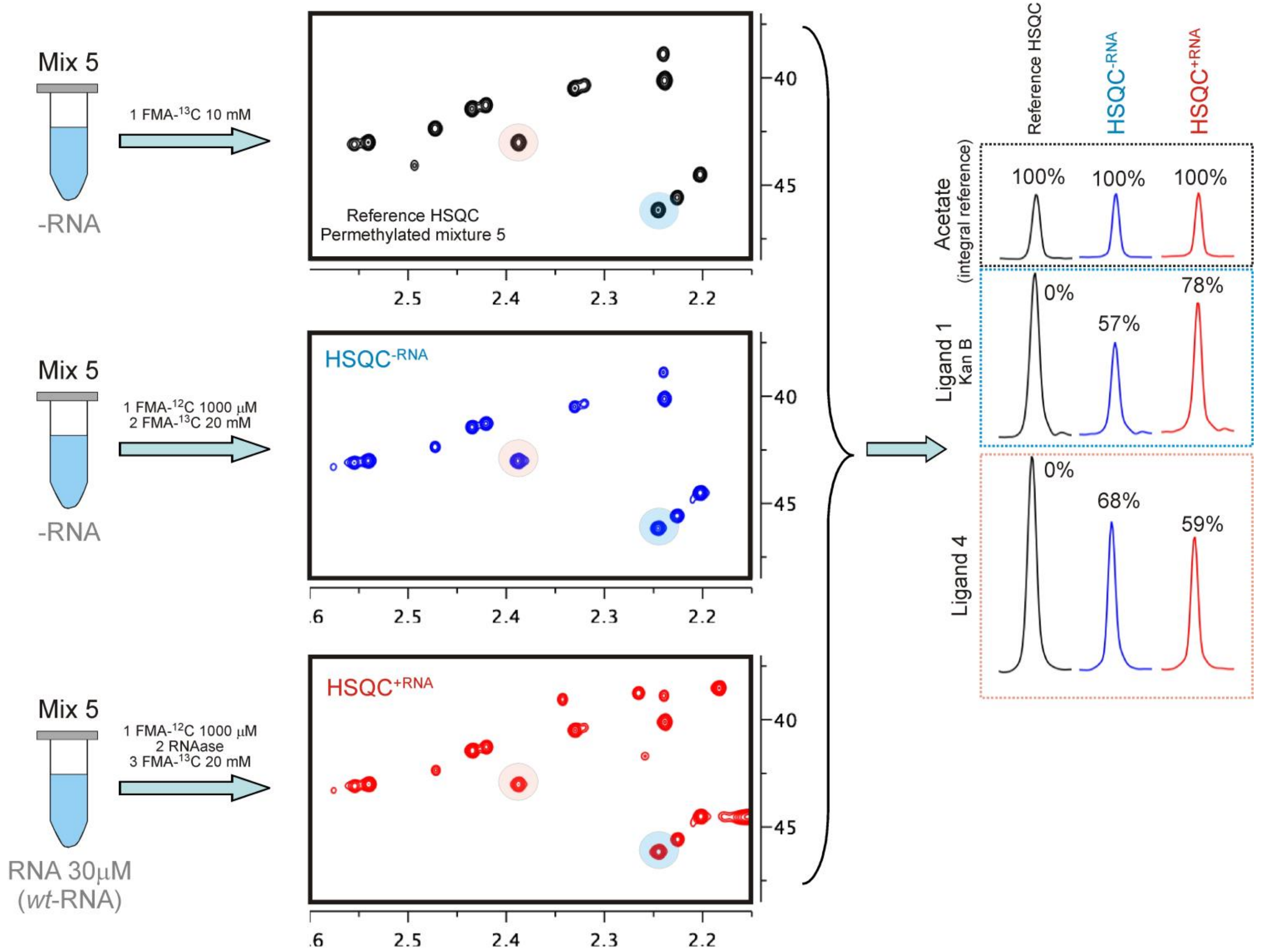

Supplementary Figure S9.- HSQC^{+RNA}, HSQC^{-RNA} and HSQC^{-diff} experiments (from left to right) obtained with a mixture of five poly-amine ligands (mix 5), employing the wild-type ribosomal A-Site as receptor and four different concentrations of unlabeled formaldehyde in step 1 of the protocol. In all cases, HSQC^{-diff} data sets are dominated by signals from the best per-*N*-methylated ligand (compound **1**). Buffer impurities are labeled with an asterisk.

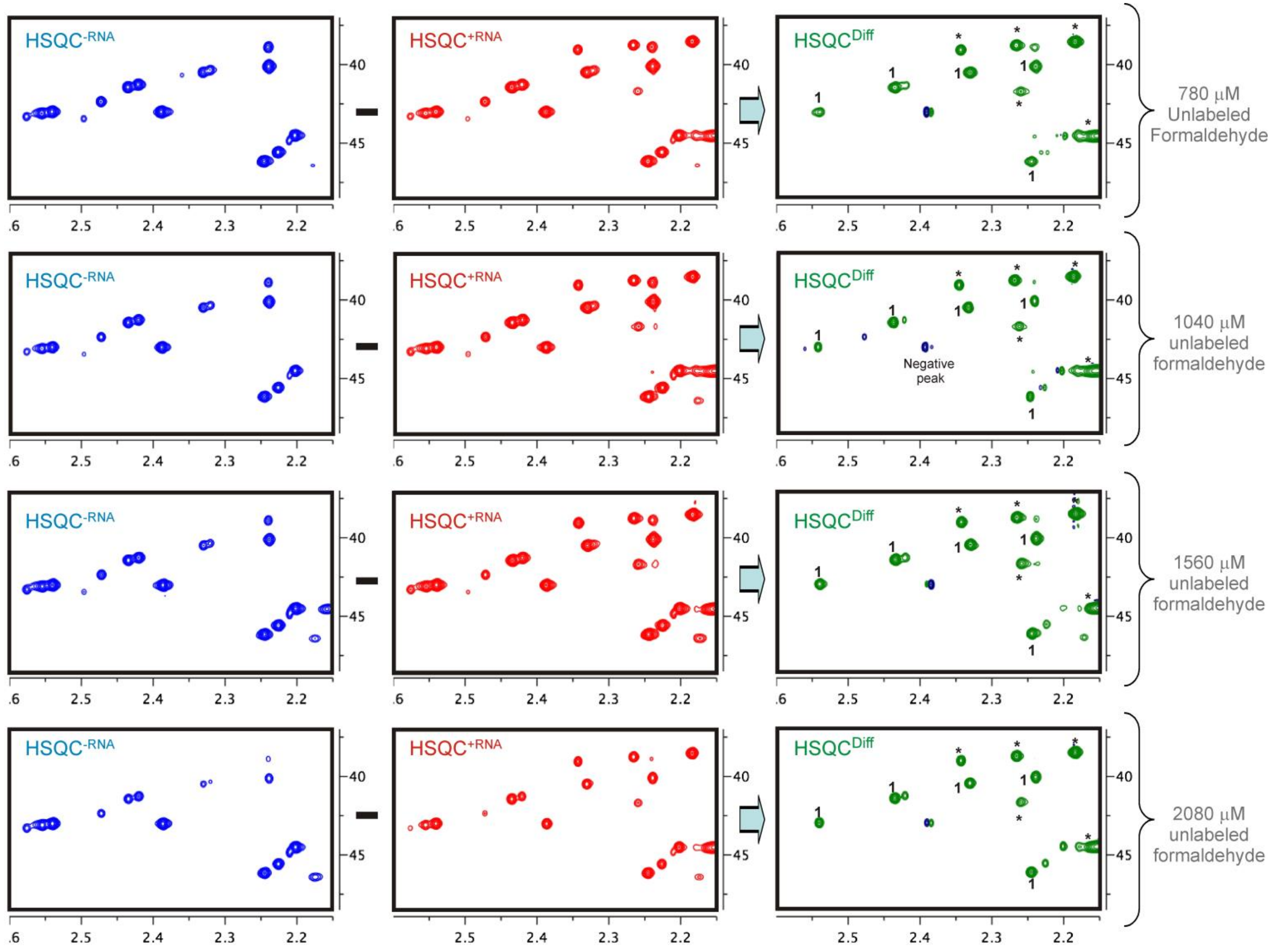

Supplementary Figure S10.- Difference HSQC experiments (HSQC^{diff}) obtained after applying our screening protocol to the mixture represented in figure S6 (reference spectra shown in the middle of the figure), employing the wild-type ribosomal A-Site as receptor and different concentrations of unlabeled formaldehyde to *delete* weak binders in step 1 of the protocol (see the experimental section). Cross peaks are assigned to the different *N*-methylated ligands according to the color code indicated in Figure S6. In all cases, the obtained results are consistent with a major protection of ligand **1** by the RNA fragment. Minor protection effects are also detected for ligand **6**. Buffer impurities are indicated with an asterisk.

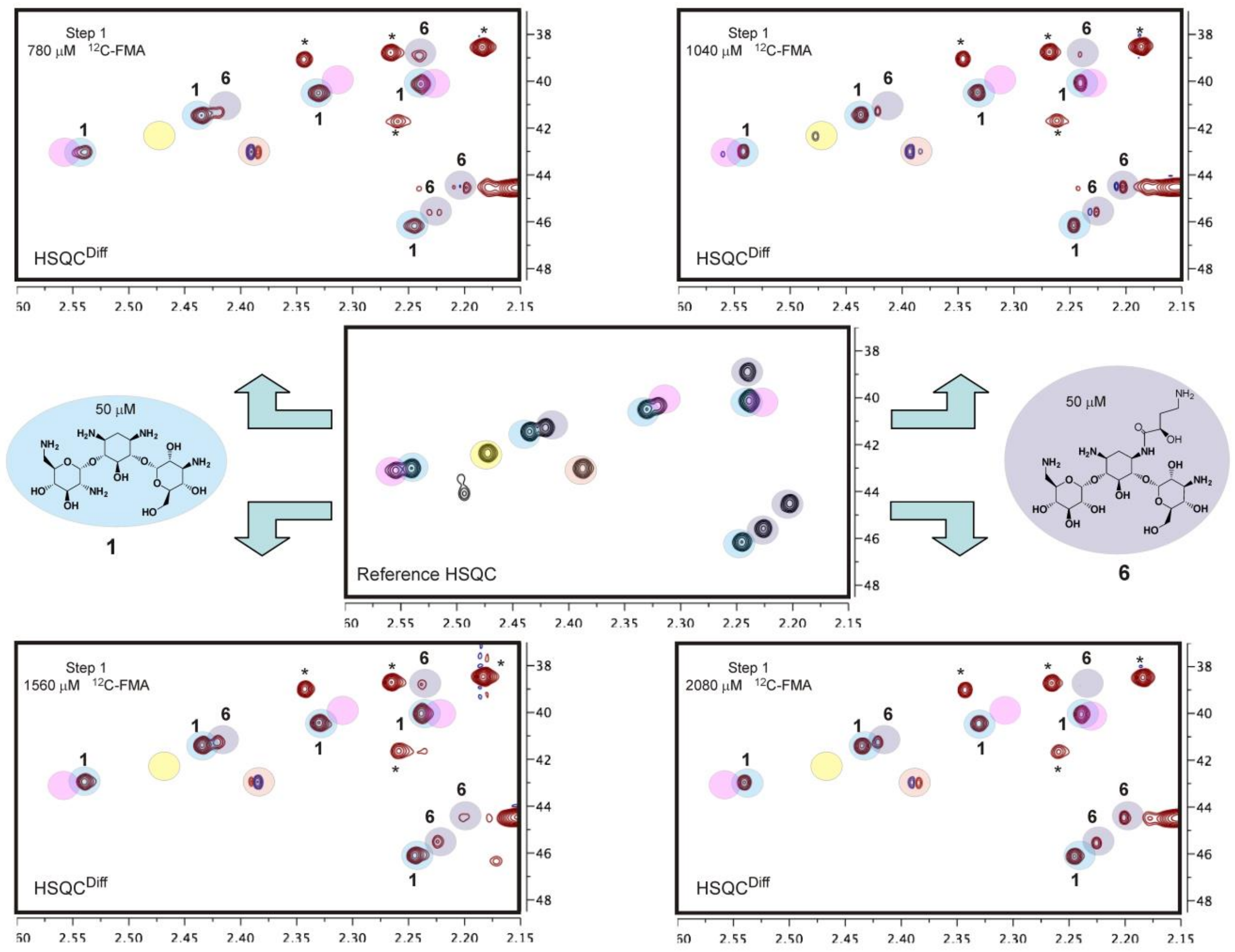

Supplementary Figure S11.- Difference HSQC experiments (HSQC^{diff}) obtained with the ligand mixture represented in figure S6 (herein referred to as mix 5) and the ribosomal A-Site as receptor, employing the standard protocol described in the experimental section (top) and a slightly modified version (bottom). In this latter case, unlabeled and labeled formaldehyde employed in steps 1 and 3, respectively, have been swapped. The resulting HSQC^{diff} data set still allows a straightforward identification of the best ligand, which in this case reveals protection effects through intense negative cross-peaks (shown in blue). Methylated buffer impurities are indicated with an asterisk.

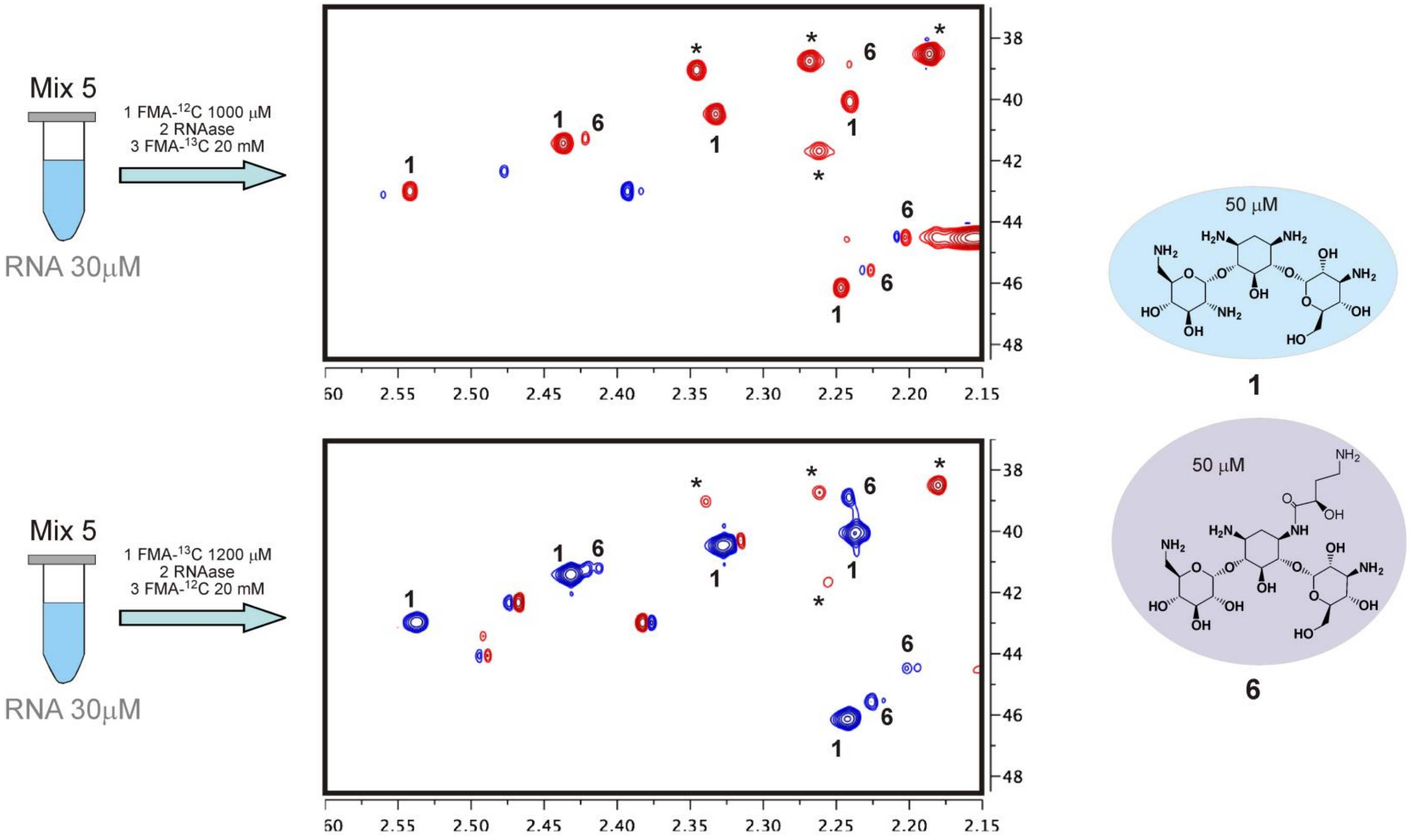

Supplementary Figure S12.- Reference HSQC experiments acquired for per-*N*-methylated derivatives **2** and **3**, together with that corresponding to mixture 3 (see Fig. 5) are represented in black. The HSQC^{diff} data set obtained after applying our screening protocol to mixture 3, employing the ribosomal A-Site as receptor is shown in red (right-bottom corner of the Figure). Methylated buffer impurities are indicated with an asterisk.

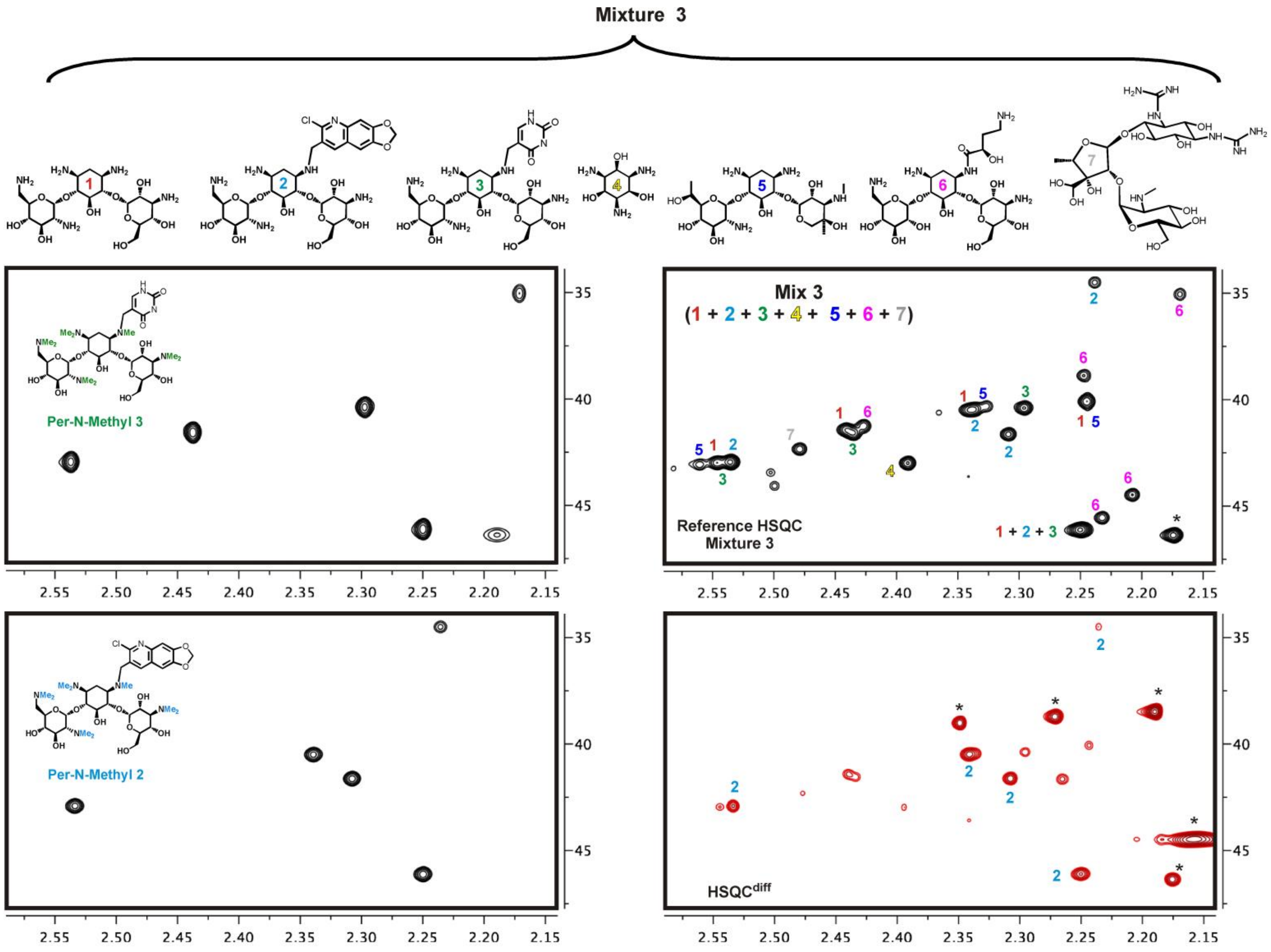

Supplementary Figure S13.- Protection assay carried out employing the duplex fragment shown on the left and a ligand library formed by 53 aminated derivatives (Figure 5). HSQC^{+DNA} (top) and HSQC^{-DNA} (bottom) spectra with identical thresholds, varying from left to right, are shown. Some examples of signals whose intensities in the HSQC^{-DNA} spectra (bottom) are larger than or identical to those exhibited in the HSQC^{+DNA} (that is not showing protection by the duplex fragment) are highlighted with a cyan circle.

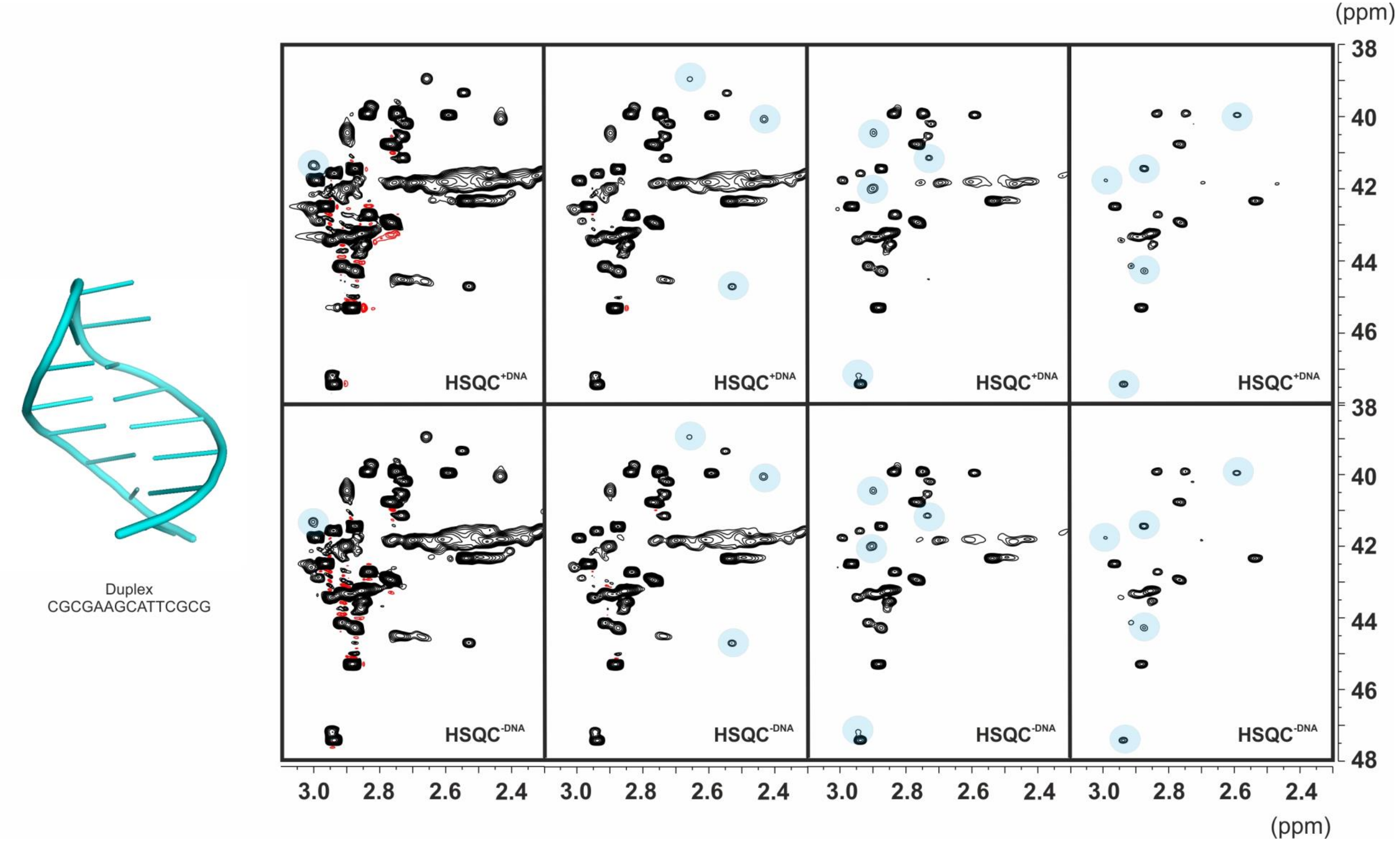

Supplementary Figure S14.- 1D STD experiment performed with a simple mixture of 10 derivatives (1-2 mM each) in the presence of the model quadruplex-duplex junction (QDJ 50 μM).

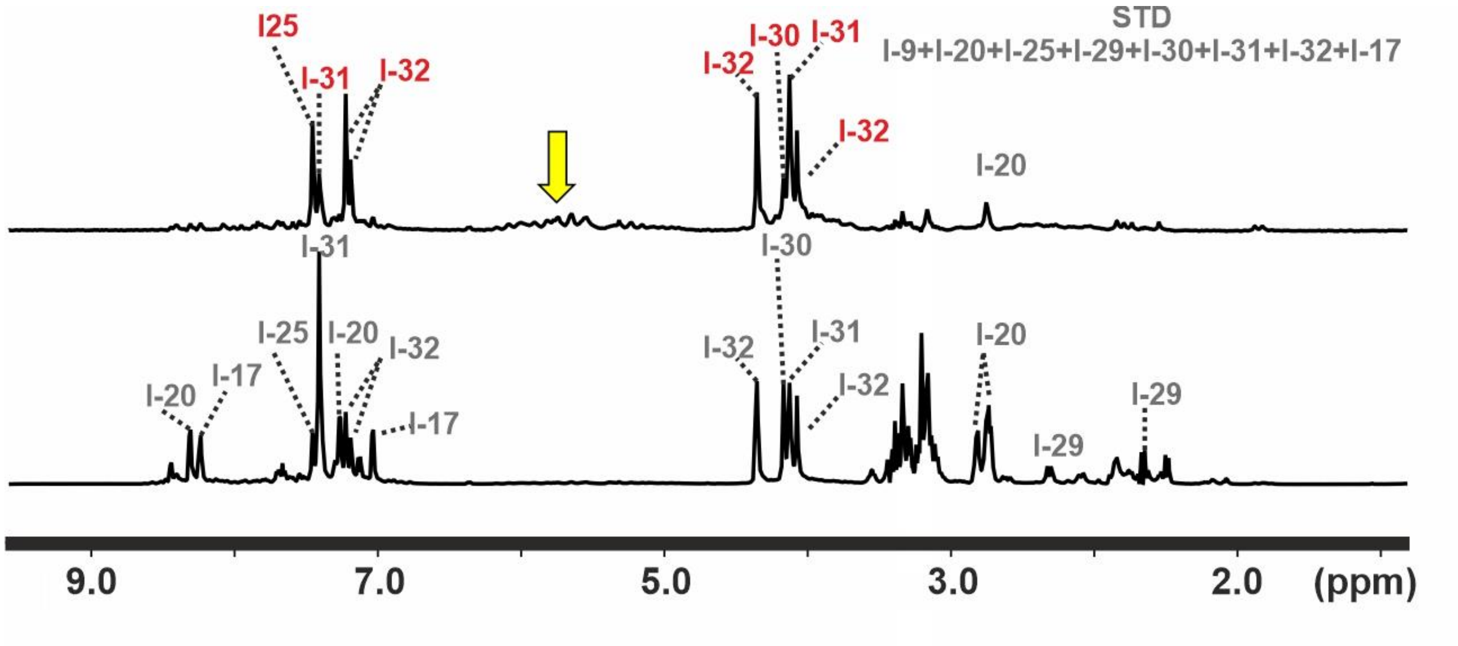

Supplementary Figure S15.- Protection assay carried out employing the QDJ1 fragment shown on the left and a ligand library formed by 53 aminated derivatives (Figure 5). HSQC^{+DNA} (top) and HSQC^{-DNA} (bottom) spectra with identical thresholds, varying from left to right, are shown. Some examples of signals whose intensities in the HSQC^{-DNA} spectra (bottom) are larger than or identical to those exhibited in the HSQC^{+DNA} (that is not showing protection by the duplex fragment) are highlighted with a cyan circle. Signals whose intensities in the HSQC^{-DNA} spectra (bottom) are lower than that exhibited in the HSQC^{+DNA} (that is showing a protection by the DNA fragment) are highlighted with a red circle.

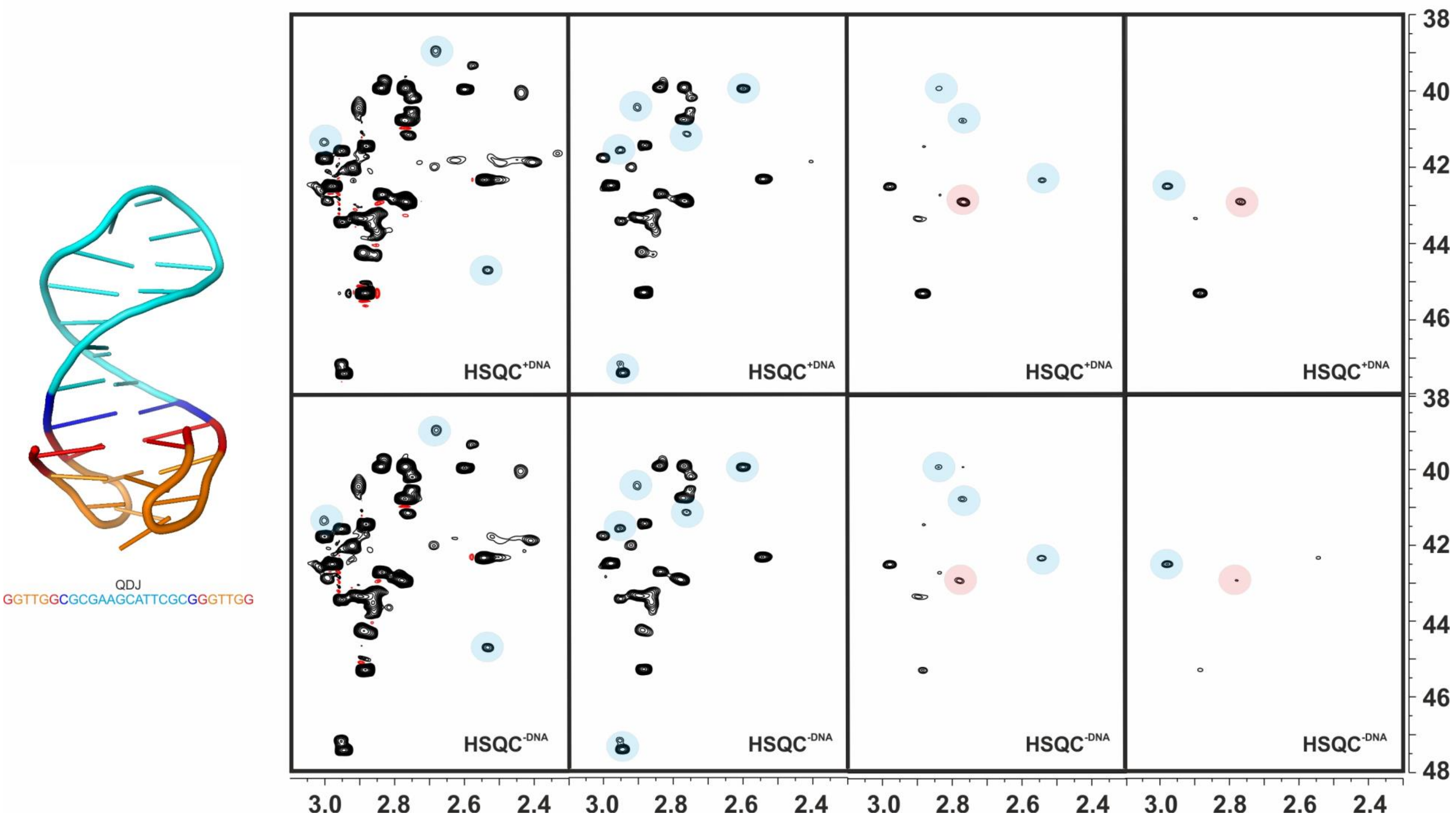

Supplementary Figure S16.- Protection assay carried out employing the HIV-LTIII fragment shown on the left and a ligand library formed by 53 aminated derivatives (Figure 5). HSQC^{+DNA} (top) and HSQC^{-DNA} (bottom) spectra with identical thresholds, varying from left to right, are shown. Some examples of signals whose intensities in the HSQC^{-DNA} spectra (bottom) are larger than or identical to those exhibited in the HSQC^{+DNA} (that is not showing protection by the duplex fragment) are highlighted with a cyan circle. Signals whose intensities in the HSQC^{-DNA} spectra (bottom) are lower than that exhibited in the HSQC^{+DNA} (that is showing a protection by the DNA fragment) are highlighted with a red circle.

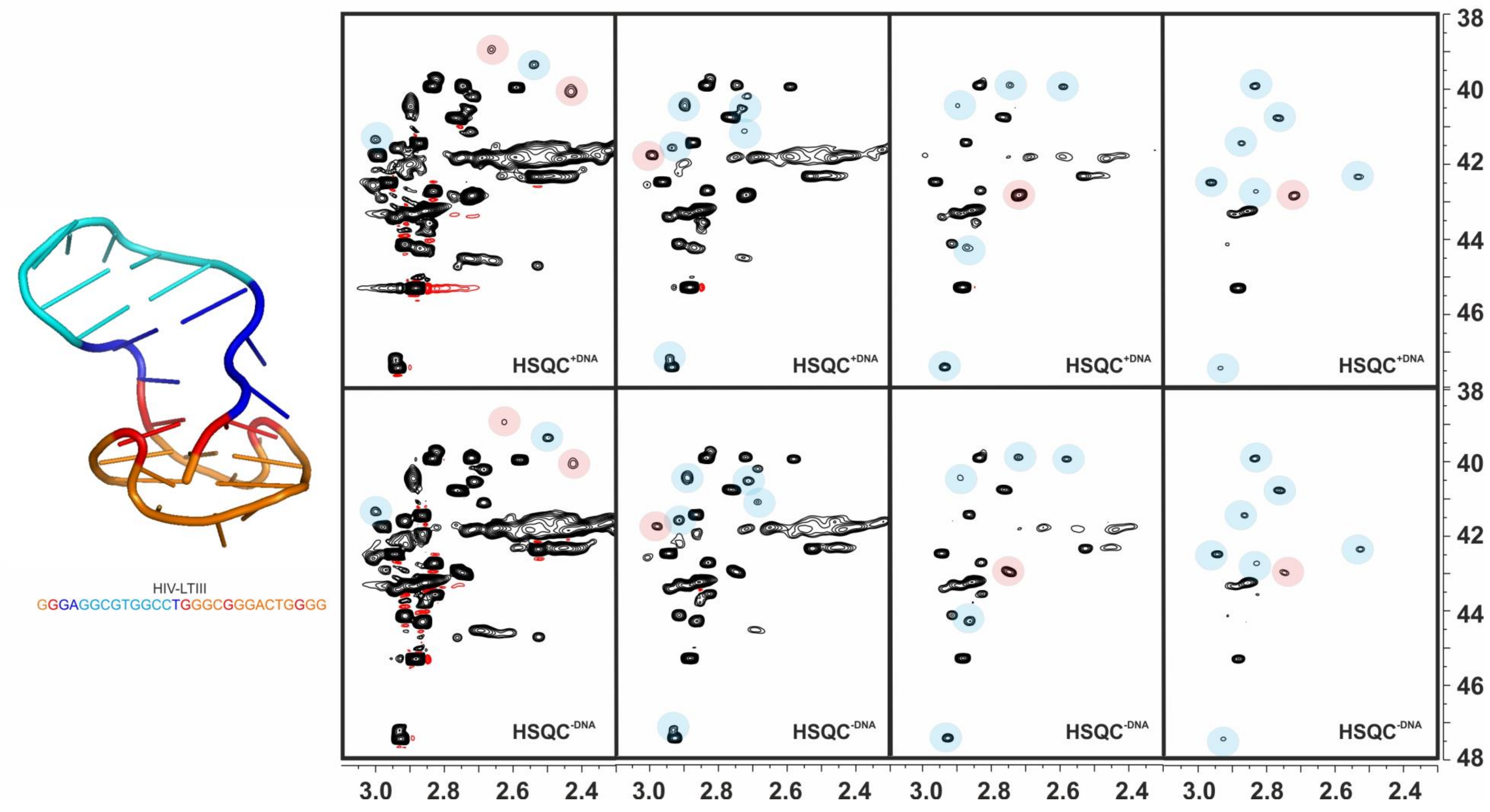

Supplementary Figure S17.- 1D STD experiment performed with a simple mixture of 6 derivatives (1-2 mM each) in the presence of the model TWJ DNA (50 μM)

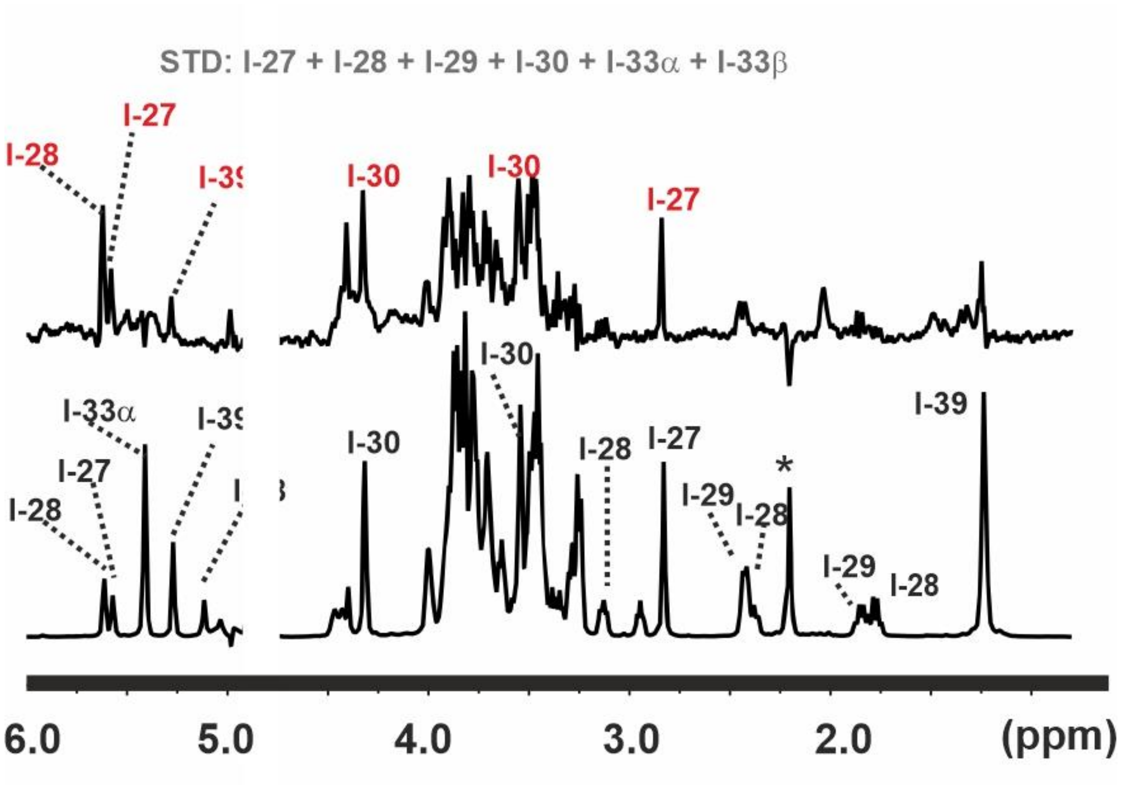

Supplementary Figure S18.- 1D-NMR titration experiments performed with ligand **l-28** and the TWJ DNA fragment (imino region displayed). Most affected imino signals are labelled

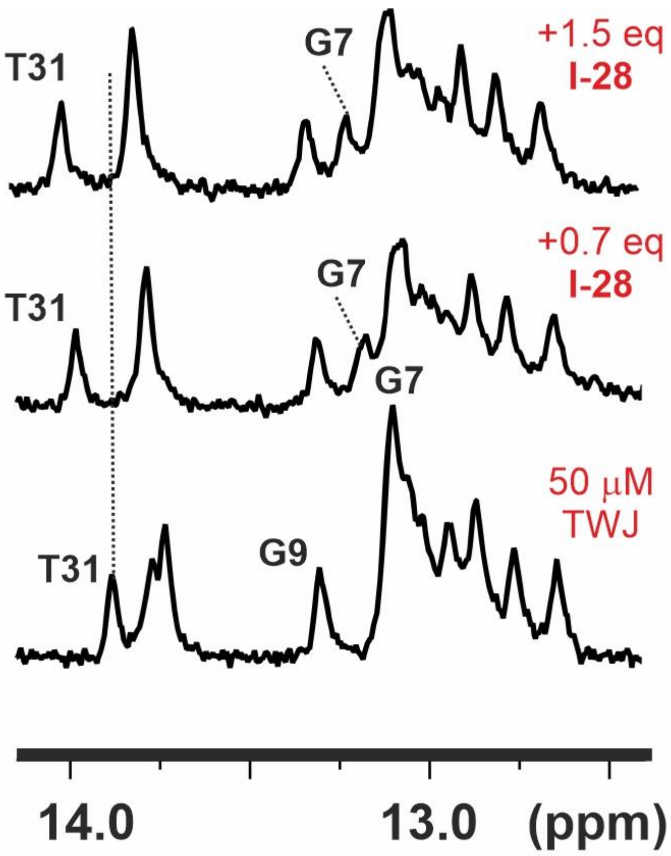

Supplementary Figure S19.- A schematic representation of the simulated assays is shown on the upper part of the Figures (a and b). They comprised a DNA/RNA receptor, an *N*-methylating reagent and two competing ligands (**L1** and **L2**), with binding affinities, either (a) $K_{b1}=10^{6}$ $M^{-1}/K_{b2}=5 \times 10^{5}$ M^{-1} or (b) $K_{b1}=10^{6}$ $M^{-1}/K_{b2}=2.5 \times 10^{5}$ M^{-1}. Concentrations for the ligands, receptor and the *N*-methylating reagent were set to 50 μM, 30 μM and 100 μM, respectively. As a first approximation, complexed ligands were assumed to be fully protected by the nucleic acid receptor, and therefore unreactive. Employing the methodology outlined in the supplementary experimental section (on page S3), relative intensities of the $HSQC^{+DNA/RNA}$ and $HSQC^{-DNA/RNA}$ cross-peaks were evaluated for both library components and are represented at the bottom of the figure (a and b). In both cases (a and b) the considered scenario favors detection of the strongest binder over the second-best ligand.

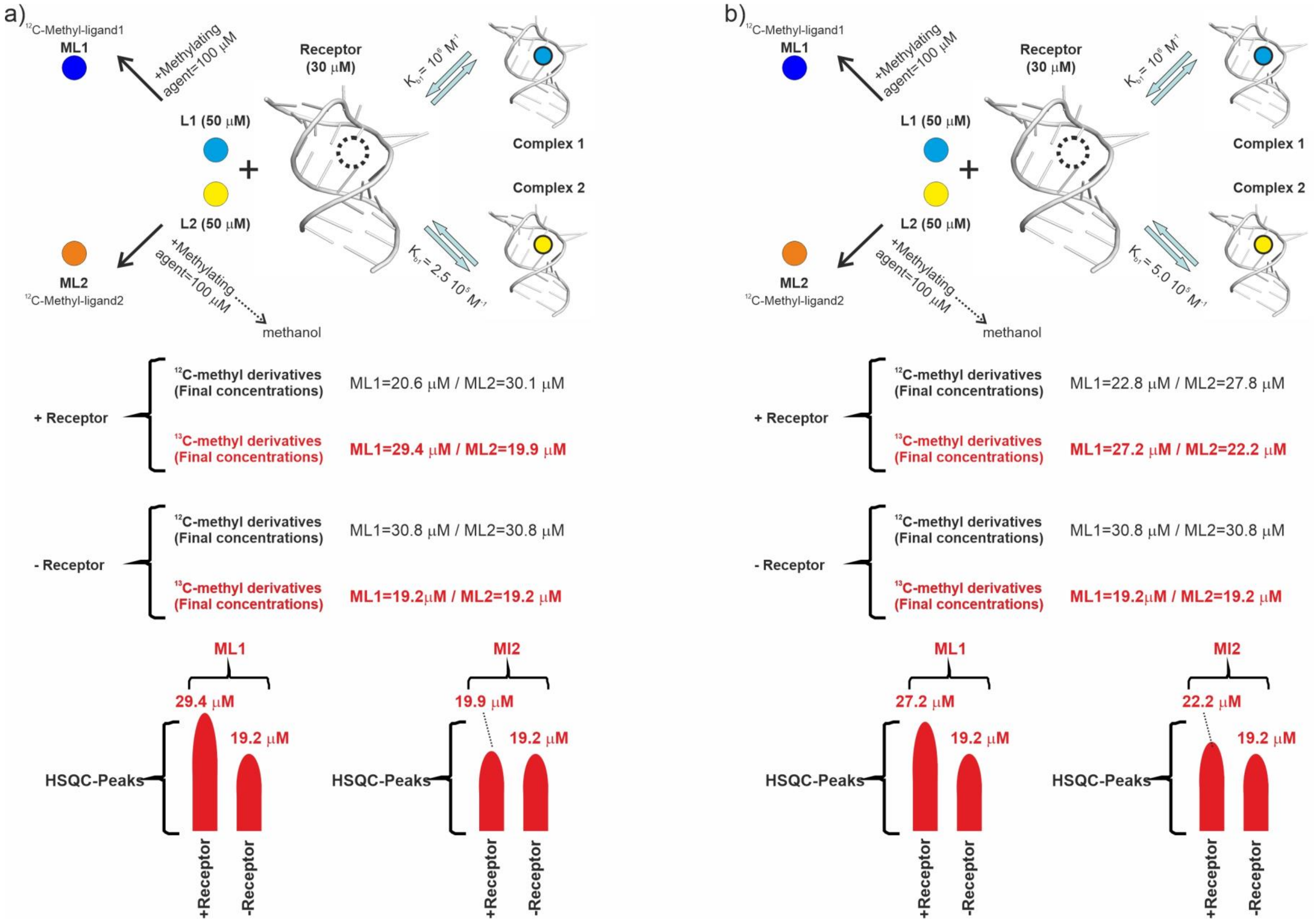
a)
^{12}C-Methyl-ligand1
ML1
+Methylating agent=100 μM
Receptor (30 μM)
K_{b1}= 10^6 M^{-1}
Complex 1
L1 (50 μM)
+
L2 (50 μM)
Complex 2
K_{b1} = 2.5 10^5 M^{-1}
ML2
^{12}C-Methyl-ligand2
+Methylating agent=100 μM
methanol
+ Receptor
^{12}C-methyl derivatives (Final concentrations)
ML1=20.6 μM / ML2=30.1 μM
^{13}C-methyl derivatives (Final concentrations)
ML1=29.4 μM / ML2=19.9 μM
- Receptor
^{12}C-methyl derivatives (Final concentrations)
ML1=30.8 μM / ML2=30.8 μM
^{13}C-methyl derivatives (Final concentrations)
ML1=19.2μM / ML2=19.2 μM
ML1
29.4 μM
19.2 μM
HSQC-Peaks
+Receptor
-Receptor
Ml2
19.9 μM
19.2 μM
HSQC-Peaks
+Receptor
-Receptor
b)
^{12}C-Methyl-ligand1
ML1
+Methylating agent=100 μM
Receptor (30 μM)
K_{b1}= 10^6 M^{-1}
Complex 1
L1 (50 μM)
+
L2 (50 μM)
Complex 2
K_{b1} = 5.0 10^5 M^{-1}
ML2
^{12}C-Methyl-ligand2
+Methylating agent=100 μM
methanol
+ Receptor
^{12}C-methyl derivatives (Final concentrations)
ML1=22.8 μM / ML2=27.8 μM
^{13}C-methyl derivatives (Final concentrations)
ML1=27.2 μM / ML2=22.2 μM
- Receptor
^{12}C-methyl derivatives (Final concentrations)
ML1=30.8 μM / ML2=30.8 μM
^{13}C-methyl derivatives (Final concentrations)
ML1=19.2μM / ML2=19.2 μM
ML1
27.2 μM
19.2 μM
HSQC-Peaks
+Receptor
-Receptor
Ml2
22.2 μM
19.2 μM
HSQC-Peaks
+Receptor
-Receptor

Supplementary Figure S20.- A schematic representation of the simulated scenario is shown on the upper part of the Figure. They comprised a DNA/RNA receptor, an *N*-methylating reagent and two competing ligands (**L1** and **L2**), with binding affinities $K_{b1}=10^6$ M^{-1}/$K_{b2}=5$ x 10^5 M^{-1}. Concentrations for the ligands, receptor and the *N*-methylating reagent were set to 50 μM, 30 μM and 100 μM, respectively. Complexed ligands were considered reactive, being the protection factors **PF1** and **PF2**, 5 and 2.5, respectively. Employing the methodology outlined in the supplementary experimental section (on page S3), relative intensities of the $HSQC^{+DNA/RNA}$ and $HSQC^{-DNA/RNA}$ cross-peaks were evaluated for both ligands and are represented at the bottom of the figure. The considered scnario favors detection of the strongest binder over the second-best ligand.

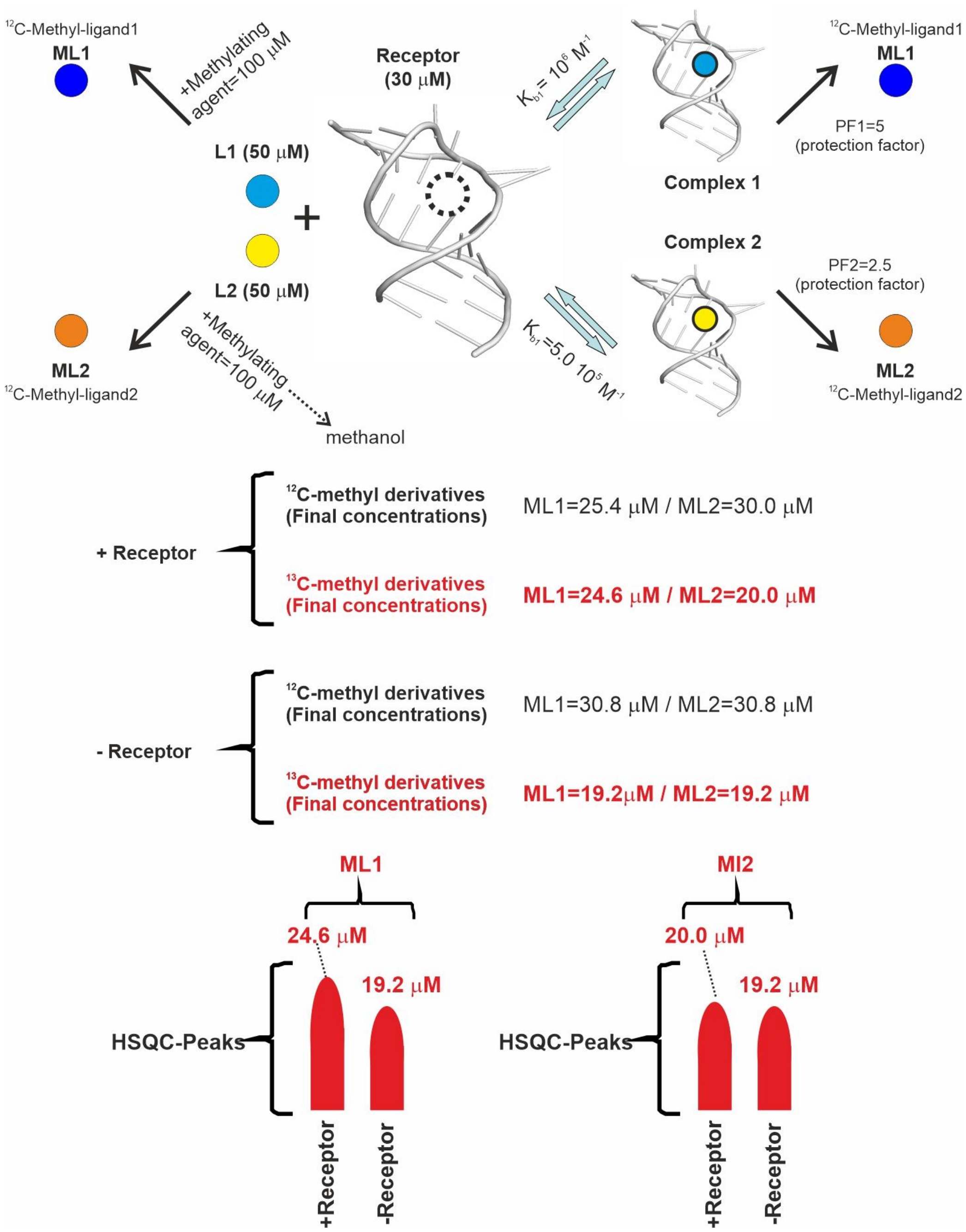
12C-Methyl-ligand1
ML1
+Methylating agent=100 μM
Receptor (30 μM)
Kb1 = 10^6 M^-1
L1 (50 μM)
L2 (50 μM)
Complex 1
Complex 2
PF1=5 (protection factor)
PF2=2.5 (protection factor)
ML2
12C-Methyl-ligand2
Kb1 =5.0 10^5 M^-1
methanol
+ Receptor
12C-methyl derivatives (Final concentrations)
ML1=25.4 μM / ML2=30.0 μM
13C-methyl derivatives (Final concentrations)
ML1=24.6 μM / ML2=20.0 μM
- Receptor
12C-methyl derivatives (Final concentrations)
ML1=30.8 μM / ML2=30.8 μM
13C-methyl derivatives (Final concentrations)
ML1=19.2μM / ML2=19.2 μM
ML1
Ml2
24.6 μM
20.0 μM
19.2 μM
HSQC-Peaks
+Receptor
-Receptor

Supplementary Figure S21.- GEPASY simulations considering more complex mixtures. A schematic representation of the simulated scenario is shown on the upper part of the Figure. It comprised a DNA/RNA receptor, and *N*-methylating reagent and five derivatives (**L1-L5**), among which only one presents affinity for the nucleic acid fragment with a K_{b1}=5 x 10^4 M^{-1}. Concentrations for each library component, the receptor and the reagent were set to 50 μM, 30 μM and 300 μM, respectively. In addition, a protection factor of 5 was assumed for ligand **L1** (**PF1** =5). Employing the methodology outlined in the supplementary experimental section (on page S3), relative intensities of the $HSQC^{+DNA/RNA}$ and $HSQC^{-DNA/RNA}$ cross-peaks were evaluated for all the library components and are represented at the bottom of the figure.

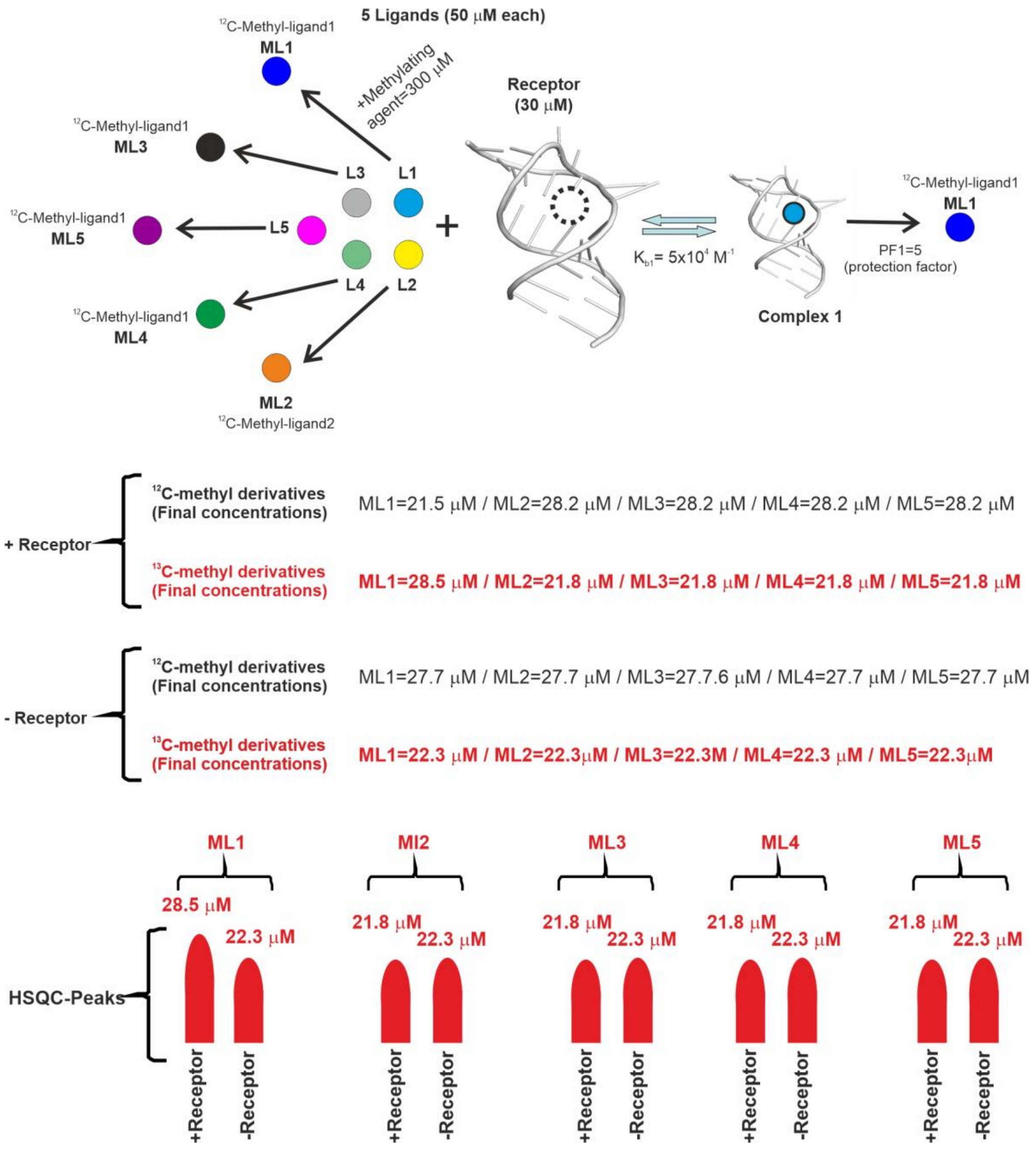

5 Ligands (50 μM each)
12C-Methyl-ligand1
ML1
+Methylating agent=300 μM
12C-Methyl-ligand1
ML3
L3
L1
12C-Methyl-ligand1
ML5
L5
L4
L2
12C-Methyl-ligand1
ML4
ML2
12C-Methyl-ligand2
Receptor (30 μM)
+
Kb1= 5x10^4 M^-1
Complex 1
12C-Methyl-ligand1
ML1
PF1=5 (protection factor)
+ Receptor
12C-methyl derivatives (Final concentrations)
ML1=21.5 μM / ML2=28.2 μM / ML3=28.2 μM / ML4=28.2 μM / ML5=28.2 μM
13C-methyl derivatives (Final concentrations)
ML1=28.5 μM / ML2=21.8 μM / ML3=21.8 μM / ML4=21.8 μM / ML5=21.8 μM
- Receptor
12C-methyl derivatives (Final concentrations)
ML1=27.7 μM / ML2=27.7 μM / ML3=27.7.6 μM / ML4=27.7 μM / ML5=27.7 μM
13C-methyl derivatives (Final concentrations)
ML1=22.3 μM / ML2=22.3μM / ML3=22.3M / ML4=22.3 μM / ML5=22.3μM
ML1
Ml2
ML3
ML4
ML5
28.5 μM
22.3 μM
21.8 μM
22.3 μM
21.8 μM
22.3 μM
21.8 μM
22.3 μM
21.8 μM
22.3 μM
HSQC-Peaks
+Receptor
-Receptor
+Receptor
-Receptor
+Receptor
-Receptor
+Receptor
-Receptor
+Receptor
-Receptor

Supplementary Figure S22.- Final GEPASY simulations were performed with the five-component library mixture. A schematic representation of this model is shown on the upper part of the Figure. It comprised a DNA/RNA receptor, the *N*-methylating reagent and five derivatives (**L1-L5**), two of them (**L1** and **L2**) displaying affinity for the nucleic acid fragment with $K_{b1}=10^6$ M^{-1} and $K_{b2}=5$ x 10^5 M^{-1}. Concentrations for the library components, the receptor, and the reagent were set to 50 μM, 30 μM and 300 μM, respectively. Protection factors of 5 and 2.5 were considered for ligand **L1** and **L2** respectively (**PF1** =5 and **PF2=2.5**). Employing the methodology outlined in the supplementary experimental section (on page S3), relative intensities of the $HSQC^{+DNA/RNA}$ and $HSQC^{-DNA/RNA}$ cross-peaks were evaluated for all the library components and are represented at the bottom of the figure. According to our analysis derivative **L1** can be readily identified as the best binder.

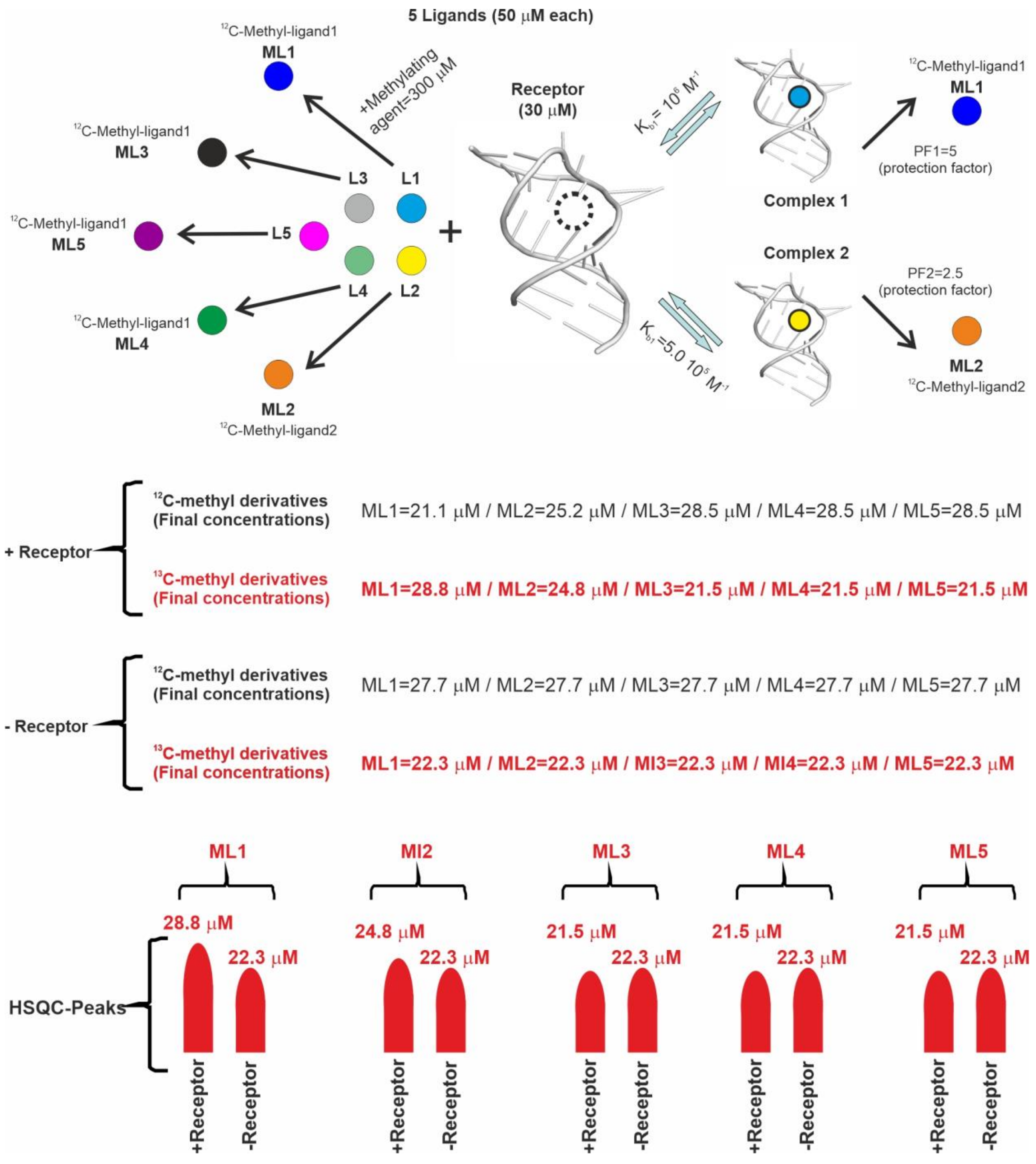

5 Ligands (50 μM each)
12C-Methyl-ligand1
ML1
+Methylating agent=300 μM
12C-Methyl-ligand1
ML3
L3
L1
12C-Methyl-ligand1
ML5
L5
L4
L2
12C-Methyl-ligand1
ML4
ML2
12C-Methyl-ligand2
+
Receptor (30 μM)
Kb1 = 10^6 M^-1
Kb1 =5.0 10^5 M^-1
Complex 1
Complex 2
12C-Methyl-ligand1
ML1
PF1=5 (protection factor)
PF2=2.5 (protection factor)
ML2
12C-Methyl-ligand2
+ Receptor
12C-methyl derivatives (Final concentrations)
ML1=21.1 μM / ML2=25.2 μM / ML3=28.5 μM / ML4=28.5 μM / ML5=28.5 μM
13C-methyl derivatives (Final concentrations)
ML1=28.8 μM / ML2=24.8 μM / ML3=21.5 μM / ML4=21.5 μM / ML5=21.5 μM
- Receptor
12C-methyl derivatives (Final concentrations)
ML1=27.7 μM / ML2=27.7 μM / ML3=27.7 μM / ML4=27.7 μM / ML5=27.7 μM
13C-methyl derivatives (Final concentrations)
ML1=22.3 μM / ML2=22.3 μM / Ml3=22.3 μM / Ml4=22.3 μM / ML5=22.3 μM
ML1
Ml2
ML3
ML4
ML5
28.8 μM
22.3 μM
24.8 μM
22.3 μM
21.5 μM
22.3 μM
21.5 μM
22.3 μM
21.5 μM
22.3 μM
HSQC-Peaks
+Receptor
-Receptor
