# Supplementary material for: Binding-driven reactivity attenuation enables NMR identification of selective drug candidates for nucleic acid targets
Source: Commun Chem. 2022 Oct 27;5:137. doi: 10.1038/s42004-022-00755-8 (PMC9814457; doi:10.1038/s42004-022-00755-8)
Supplement: Supplementary file 1 — Asensio_PR File [file 42004_2022_755_MOESM1_ESM.pdf]

nature portfolio

Peer Review File

Binding-driven reactivity attenuation enables NMR identification of selective drug candidates for nucleic acid targets

Open Access This file is licensed under a Creative Commons Attribution 4.0 International License, which permits use, sharing, adaptation, distribution and reproduction in any medium or format, as long as you give appropriate credit to the original author(s) and the source, provide a link to the Creative Commons license, and indicate if changes were made. In the cases where the authors are anonymous, such as is the case for the reports of anonymous peer reviewers, author attribution should be to 'Anonymous Referee' followed by a clear attribution to the source work. The images or other third party material in this file are included in the article's Creative Commons license, unless indicated otherwise in a credit line to the material. If material is not included in the article's Creative Commons license and your intended use is not permitted by statutory regulation or exceeds the permitted use, you will need to obtain permission directly from the copyright holder. To view a copy of this license, visit http://creativecommons.org/licenses/by/4.0/.

Reviewers' comments:

Reviewer #1 (Remarks to the Author):

The manuscript by Díaz-Casado et al presents a novel NMR method for screening nucleic-acid-binding small molecules. It has become increasingly clear that nucleic acids, including DNA and RNA, can be important targets for developing therapeutics for human diseases. Hence it is of great interests to develop robust and effective methods for screening small molecules that specifically recognize nucleic acids. In the present work, the authors developed a strategy based on ligand reactivity for NMR screening of small molecules that are protected from chemical modifications upon binding to nucleic-acid targets. The authors first presented their concept using kanamycin-B and ribosomal A-site RNA fragment as a model system. Subsequently, they demonstrated the reactivity-based method on A-site RNA using small libraries composed of 2 to 7 small molecules. Lastly, the authors built a library composed of 53 mono- and poliaminated derivatives and applied their method to screen small-molecule binders to three DNA systems, including quadruplex-duplex junction (QDJ), HIV-LTIII fragment, and three-way junction (TWJ). While the present approach is limited to small molecules with reactive amine groups, it does provide an innovative concept for screening nucleic acid binders and should be of great interests to the field of nucleic acid drug discovery. Hence, I highly recommend publication of this work with a few minor questions and suggestions for the authors to consider in their revised manuscript.

1. One main question I had is that the current approach seems to bias in identifying the strongest binder among the library and is less sensitive to molecules with comparable but slightly weaker binding affinities. For example, the authors showed previously that QDJ binds I-31 and I-32 with affinities of 1.2 uM and 2.3 uM, respectively. While it is satisfying that the present approach successfully identified the tightest binder, I-31, it is surprising that I-32, which would be considered as a good binder, was not detected in the assay. Similar phenomena can also be seen in the binding between A-site and kanamycin-B (molecule 1) and its aromatic derivative (molecule 2). Could the authors elaborative on these observations? Could it be due to the experimental setup of the relative concentrations between target and small molecules?

2. The author did a nice benchmark to analyze the effect of mono-valent Na+. It is known that Mg2+ plays important roles in the folding and structure of nucleic acids. Can the authors comment on the applicability of their approach for systems that require Mg2+?

3. The authors have provided binding affinities for small molecules that bind A-site, QDJ, and HIV-LTIII. For the completeness, the authors might also want to report binding affinities for I-28 and I-32 to TWJ.

Reviewer #2 (Remarks to the Author):

This is an interesting novel approach to ligand based screening: How do protection factors relate to Kds? I see protection factors up to around 15. Is it conceivable that there are more intricate effects than steric hindrance from the reaction that might even accelerate the methylation in the bound form, i.e. the polynucleotide target could be catalytic.
For the experiment with the aminoglycosides, the concentration of RNA fragment is 50 mikromolar, but of the aminoglycosides 40 mikromolar. This is also true for Fig. 3. In that figure 3 ligands have protection factors of around 20(Pf). Could the authors explain how this is plausible. Since the Pfs are approximately the same for all 3 ligands, then for each ligand there would be maximum 50/3 mikromolar RNA available = 16 mikromolar is bound to RNA. This would mean that at a given time more than half of the ligand is not RNA bound. Assuming that the bound form is fully protected from the reaction, the free form however not, would allow for Pfs of 2, but not 20. Does this indicate that

also surface interactions of RNA with the ligand (also observed by STD and tranfser NOE) will slow down the reactivity? If that was the case, then the difference to STD is less since then also attenuation is feasible by binding to more shallow binding sites.
There are funny phases (negative (red) peaks next to black (positive) in Fig. 7c right most.
What if the methylated small molecule binds more strongly than the non-methylated? Would that endanger the approach?
The example shown indicate that non specific binding or binding to flat binding pockets which is picked up by STD, but not by the reactivity method discussed here. Yet, this is rather presented as an interpretation than as a plausible fact.

COMMSCHEM-22-0249

Reviewer 1

- "One main question I had is that the current approach seems to bias in identifying the strongest binder among the library and is less sensitive to molecules with comparable but slightly weaker binding affinities. For example, the authors showed previously that QDJ binds I-31 and I-32 with affinities of 1.2 uM and 2.3 uM, respectively. While it is satisfying that the present approach successfully identified the tightest binder, I-31, it is surprising that I-32, which would be considered as a good binder, was not detected in the assay. Similar phenomena can also be seen in the binding between A-site and kanamycin-B (molecule 1) and its aromatic derivative (molecule 2). Could the authors elaborative on these observations? Could it be due to the experimental setup of the relative concentrations between target and small molecules?"

Regarding the assays performed with ***simple mixtures*** formed by just 2-8 derivatives (such as those represented in Figure 4) the referee is right: the employed experimental protocol favors detection of the stronger binder over weaker competitors. The reason is that in the absence of DNA/RNA the unlabeled formaldehyde employed in the first "*deleting*" step is consumed by all the mixture components, according to their respective reactivities. Apparent reactivities are altered in the presence of the nucleic acid receptor, which implies not only that the most protected derivative consumes less ^{12}C-formaldehyde but also that weaker binders and non-binders consume a proportionally larger fraction of this reagent. In simple terms, t*he more "protected" a given derivative is, the more "deleted" the rest of the library components become*. This magnifies the differences among compounds (as proved by numerical simulations performed with the program GEPASI) and could explain the behavior exhibited by **kanamycin-B** and its aromatic derivative **2** in the experiments described in Figure 4. Understandably, with libraries of larger complexity this effect becomes less important and should be minor for the 53-component mixture employed in our final screening assays (which also includes derivatives **I-32** and **I31**). Yet, it is important to emphasize that the magnitude of the protection effects measured for the different mixture components is not strictly proportional to their binding affinity, as other factors are also at play. Thus, the reactivity changes detected for any given derivative depends on its bound molar fraction but also on the protection factor (Pf) for each reactive function, that is by how much its reactivity is attenuated within the receptor binding pocket. Interestingly, both factors are expected to be correlated and could act cooperatively to facilitate the preferred detection of stronger over weaker binders. The reason is that, assuming identical binding sites and geometries, stronger binders are also expected to exhibit larger protection factors. This effect is nicely illustrated by the association of kanamycin to the A-site fragment at high and low ionic strength (represented in Figure 3) where the most stable complex was shown to be significantly less reactive. According to this view, in any screening scenario weaker binders are expected to present not only lower bound molar fractions but also lower protection factors. This *double penalty* could also contribute to exacerbate the protective influence detected in best versus weaker binders.

Finally, it should be noted that for complex mixtures the bound molar fraction for any given derivative might be affected by the existence of other weaker competitors, even if undetected by the reactivity assay due to their lower affinity and/or reduced protection factors. Consequently, this parameter cannot be simply inferred from its binding affinity alone which might also explain the observed differences between **I31** and **I32**.

- “The author did a nice benchmark to analyze the effect of mono-valent Na+. It is known that Mg2+ plays important roles in the folding and structure of nucleic acids. Can the authors comment on the applicability of their approach for systems that require Mg2+?”

The influence exerted by the ionic strength on the protection factors has been illustrated with the kanamycin/A-site complex employing sodium chloride (Figure 4). Our approach would be equally applicable in the presence of $MgCl_2$ or $MgSO_4$ and, in principle, the effect of these electrolytes on the assay should be analogous to that previously described; *ionic strength generally decreases the stability of the receptor/ligand complexes together with the observed protection factors*. On the other hand, It should be noted that in particular cases Mg^{2+} can also work as a ligand itself. Indeed, the presence of specific Mg^{2+} binding sites is not entirely uncommon for RNA receptors. In these cases, Mg^{2+} could compete with the library components for specific binding pockets increasing the binding affinity required for detection. These points have been clarified in the new version of the manuscript (page 7).

- “The authors have provided binding affinities for small molecules that bind A-site, QDJ, and HIV-LTIII. For the completeness, the authors might also want to report binding affinities for I-28 and I-32 to TWJ”.

Attempts were made to determine the binding affinities of **I-28** and **I-32** to the TWJ fragment by means of NMR titrations. These experiments showed that saturation of the receptor at 50 μM DNA were superior to 80% and 90% for 1:1 and 2:1 ligand/DNA ratios, respectively, which allowed us to stablish a lower limit for the affinity constants. According to the obtained results Kb values must be superior to 200000 M^{-1} for both **I-28** and **I-32**. A more precise estimation of the binding affinities would require experiments with a significantly reduced DNA concentration, something unfeasible even for high field modern spectrometers. However, titrations performed with alternative ligand mixtures under identical experimental conditions detected no significant association of any other component. In summary, the obtained results confirmed that the protection assay successfully detected the best binders present in the library and their binding affinities are at least in the 10^5 M^{-1} range. These points have been clarified in the new version of the manuscript (page 14).

Reviewer 2

- "How do protection factors relate to Kds? I see protection factors up to around 15. Is it conceivable that there are more intricate effects than steric hindrance from the reaction…?"

Protection factors reflect how much the ligand reactivity its attenuated upon association to a given receptor. Understandably, it is a complex parameter which depends on a variety of factors, the most evident being the accessibility of the reactive groups in the bound state. This dependency implies that protection factors are sensitive to the topology of the receptor binding site (buried or superficial) and the orientation of the ligand. Dynamic fluctuations of the complex might also play an important modulatory role facilitating or hindering the encounter between reactive species. Moreover, direct involvement of the reactive ligand functions in strong inter-molecular interactions, (i.e. hydrogen bonds, salt-bridges, cation/π bonds etc) would be expected to exert a largely inhibitory influence on its reactivity. Notwithstanding, subtler effects could also be at play. For example, reductive amination reactions are sensitive to the protonation state of the amino groups, which are usually significantly altered as a result of complex formation. Indeed, pKa perturbations of ligand groups have been reported for several RNA complexes. Even changes in solvation could also contribute to the observed reactivity alterations.

However, despite the inherent complexity of the topic, a significant part of the factors contributing to ligand protection is inevitably connected with the ligand affinity. Thus, buried/concave "*more protecting*" binding pockets provide an increased surface for stablishing ligand-receptor contacts than superficial/planar sites, and frequently present more strongly negative electrostatic potentials which would translate into more stable associations. Similarly, enhanced intermolecular interactions produce more stable but also less dynamic complexes which could further inhibit the ligand reactivity. Finally, participation of the ligand reactive functions in strong interactions with the DNA/RNA fragment should reciprocate in both increased protection factors and binding affinities.

Taken all these points together into consideration, protection factors and complex stability would be expected to be, to some extent, correlated. This correlation is nicely illustrated by the association of kanamycin to the A-site fragment at high and low ionic strengths (represented in Figure 3) where, having both complexes identical geometry (as proved by the protection factor profiles) the most stable complex was also significantly less reactive. Further evidence for the mentioned correlation is the fact that all the assays described in the manuscript succeeded in identifying the best binder within the employed libraries.

Some comments about this question have been incorporated in the new version of the Supporting Information (page S2).

-"Is it conceivable that….. the reaction that might even accelerate the methylation in the bound form, i.e. the polynucleotide target could be catalytic.?

Yes. Stabilization of the reaction transition state by the DNA/RNA receptor, though very unlikely, is not implausible and would translate into an enhanced reactivity for the complexed ligand. However, we would like to point out that, although the proposed methodology was originally conceived to detect *"protection effects",* it could be equally employed to reveal any change in reactivity, including catalytic influences. Thus, according to our protocol, complexed ligands *protected* by the DNA/RNA from the unlabeled

formaldehyde will incorporate larger fractions of the ^{13}C-labeled reagent after the receptor digestion, showing larger cross-peaks in the HSQC$^{+DNA/RNA}$ spectrum with respect to the reference data set HSQC$^{-DNA/RNA}$. This is the most common situation and the behavior observed in all the assays included in the manuscript. Conversely, catalysis by the DNA/RNA fragment would translate into an enhanced incorporation of unlabeled *N*-Methyl groups, implying that the corresponding signals will display significantly reduced intensities in the HSQC$^{+DNA/RNA}$ experiment. In summary, any change in reactivity, either inhibition or enhancement, could be equally revealed by comparing the HSQC$^{+DNA/RNA}$ and HSQC$^{-DNA/RNA}$ data sets. In addition, these reactivity perturbations could also be taken as evidence of binding for screening purposes. A few comments on this point have been incorporated in the new version of the manuscript (page 15).

-"For the experiment with the aminoglycosides, the concentration of RNA fragment is 50 mikromolar, but of the aminoglycosides 40 mikromolar. This is also true for Fig. 3. In that figure 3 ligands have protection factors of around 20(Pf). Could the authors explain how this is plausible. Since the Pfs are approximately the same for all 3 ligands, then for each ligand there would be maximum 50/3 mikromolar RNA available = 16 mikromolar is bound to RNA. This would mean that at a given time more than half of the ligand is not RNA bound. Assuming that the bound form is fully protected from the reaction, the free form however not, would allow for Pfs of 2, but not 20. Does this indicate that also surface interactions of RNA with the ligand (also observed by STD and tranfser NOE) will slow down the reactivity? If that was the case, then the difference to STD is less since then also attenuation is feasible by binding to more shallow binding sites."

First, we would like to apologize for the lack of clarity of Figure 3 and the confusion this seems to have caused. Figure 3a shows the protection factors measured for kanamycin in *three independent assays*, each one performed employing 50 μM RNA (either the A-site under low or high ionic strength conditions, or a mutated version of the A-site) and 40 μM aminoglycoside. Similarly, Figure 3b represents the protection factors measured for the kanamycin derivative **2** in two *independent assays,* each one employing 50 μM and 40 μM concentrations for the RNA and the aminoglycoside, respectively. These imply that the fraction of bound ligand in the experiments without ionic strength (represented in magenta in Figure 3a) were clearly superior to 95% and therefore, fully compatible with protection factors close to 20. For the new version of the manuscript we have tried to clarify this question (legend for Figure 3).

- "There are funny phases (negative (red) peaks next to black (positive) in Fig. 7c right most."

Subtraction of the HSQC$^{+DNA/RNA}$ and HSQC$^{-DNA/RNA}$ spectra provides a simple means to highlight differences between both experiments. In principle, the resulting HSQC^{Diff} data set should only include cross peaks corresponding to ligands whose reactivity has been significantly altered as a result of complexation to the nucleic acid fragment. Unfortunately, extremely minor pH differences between NMR samples (difficult to avoid from a practical perspective) can lead in particular cases to an imperfect subtraction, responsible for the slight "antiphase" look exhibited by some signals. This point has been clarified in the new legend for Figure 7. However, we would like to point out that although this effect does contribute to general appearance of the spectra, it does not

interfere with the identification of the protected ligands as shown in the reported assays. On the other hand, subtraction of the HSQC experiments is not the only method for analysis and similar information can be obtained by a detailed comparison of the $HSQC^{+DNA/RNA}$ and $HSQC^{-DNA/RNA}$ spectra as illustrated in Figures S12-S14.

- "What if the methylated small molecule binds more strongly than the non-methylated? Would that endanger the approach?"

In principle, for mono-aminated derivatives the methylated compound would act as a competitor, probably reducing the bound molar fraction for the initial derivative and the overall protective influence exerted by the receptor. Indeed, If the affinity differences between modified and unmodified components is large enough it might be hard to detect any effect at all. On the contrary, in the case of poly-aminated compounds the modified "*high affinity*" derivative would be expected to become even more shielded from further *N*-methylation processes at other sites. The reactivity of these alternative reactive positions should reflect this enhanced protective influence, which could be employed for detection purposes. Finally, we would like to point out that, in our opinion, the proposed strategy provides a novel and valuable alternative intended to complement current NMR-based screening methodologies. Understandably, as any other approach, it has advantages and limitations and for this reason combining *reactivity assays* with more conventional NMR experiments provides the best chances for success in ligand screening.

- The example shown indicate that nonspecific binding or binding to flat binding pockets which is picked up by STD, but not by the reactivity method discussed here. Yet, this is rather presented as an interpretation than as a plausible fact.

A fundamental hypothesis behind the proposed reactivity-based assay is that ligand association to buried sites or occluded binding pockets will, in most cases, induce a sharper decrease in its reactivity than non-specific electrostatic interactions, usually more superficial and highly dynamic in character. As previously mentioned, *protection* and binding affinity are to some extent correlated, implying that the most protected ligand is on many occasions the best binder as well. Several pieces of evidence in favor of this hypothesis are presented. Thus, while the reported STD or trNOE experiments identify numerous associations, our reactivity assays show that only a very limited number of them exert a measurable influence on the ligand reactivity. Moreover, these processes typically comprise buried binding pockets and involve tighter binders. Noticeably, the reactivity assays described in the manuscript have in all cases succeeded in *identifying the best ligand within the analyzed mixtures*. The existing relationship between binding mode and protection is nicely illustrated by the control experiments described in Figure 3b. Thus, mutation of the internal loop present in the A-site yields a narrower pocket which prevents a deeper penetration of the bulky derivative **2**. As expected, this translates into reduced protection factors and a more dramatic effect would be expected for even more superficial associations. Of course, the possibility that flat/superficial sites might promote tight associations cannot be entirely ruled out. Indeed, these processes could trigger a significant protective influence for interfacial ligand reactive groups. However, based on the reasoning already outlined, these associations should be uncommon.

We really would like to thank both reviewers for their thorough analyses and for their insightful comments, and we hope that the new version of the manuscript we have produced addresses all concerns.

Sincerely,

Dr. Juan Luis Asensio

REVIEWERS' COMMENTS:

Reviewer #1 (Remarks to the Author):

The responses from Díaz-Casado et al has addressed most of my comments of the original manuscript. I think their discussions to my first comment in the response letter, such as numerical simulations using GEPASI, would be very valuable to the readers and should be included in their revised manuscript. Other than this minor suggestion, I highly recommend publication of this work.

Reviewer #2 (Remarks to the Author):

all my comments are adequately addressed.
the manuscript is ready for publication.
